# Supplementary figures and images for: Functional and biochemical characterization of the Toxoplasma gondii succinate dehydrogenase complex
Source: PLoS Pathog. 2023 Dec 11;19(12):e1011867. doi: 10.1371/journal.ppat.1011867 (PMC10735183; doi:10.1371/journal.ppat.1011867)

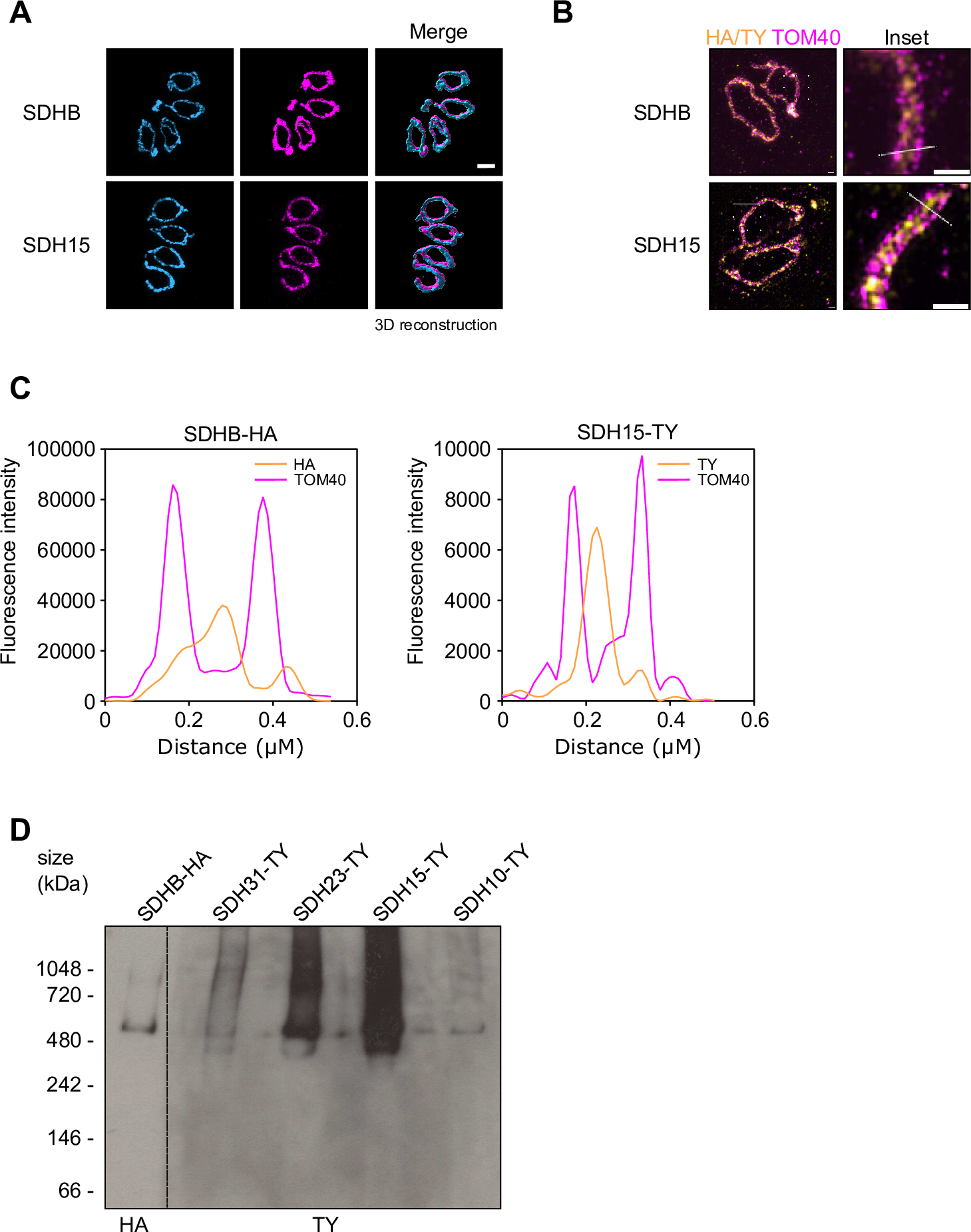

Supplement: S1 Fig — (A) Super resolution microscopy images shown as Z-stack (Left and middle panel) and 3D reconstruction (right panel) of SDHB-HA and SDH15-Ty, with the mitochondrial marker protein TOM40. Scale bar is 2 μM. (B) Ultra-expansion microscopy images from Fig 1B. Left: Merge of SDHB-HA/SDH15-Ty (orange) and TOM40 (pink) with inset area highlighted, Right: close-up of inset with white line indicating area of intensity calculation. Scale bar is 1 μM (C) Intensity plot of SDHB-HA/SDH15-Ty and TOM40 signal from part B. (D) Full immunoblot of the data shown in Fig 1C. (TIF) [file ppat.1011867.s001.tif]

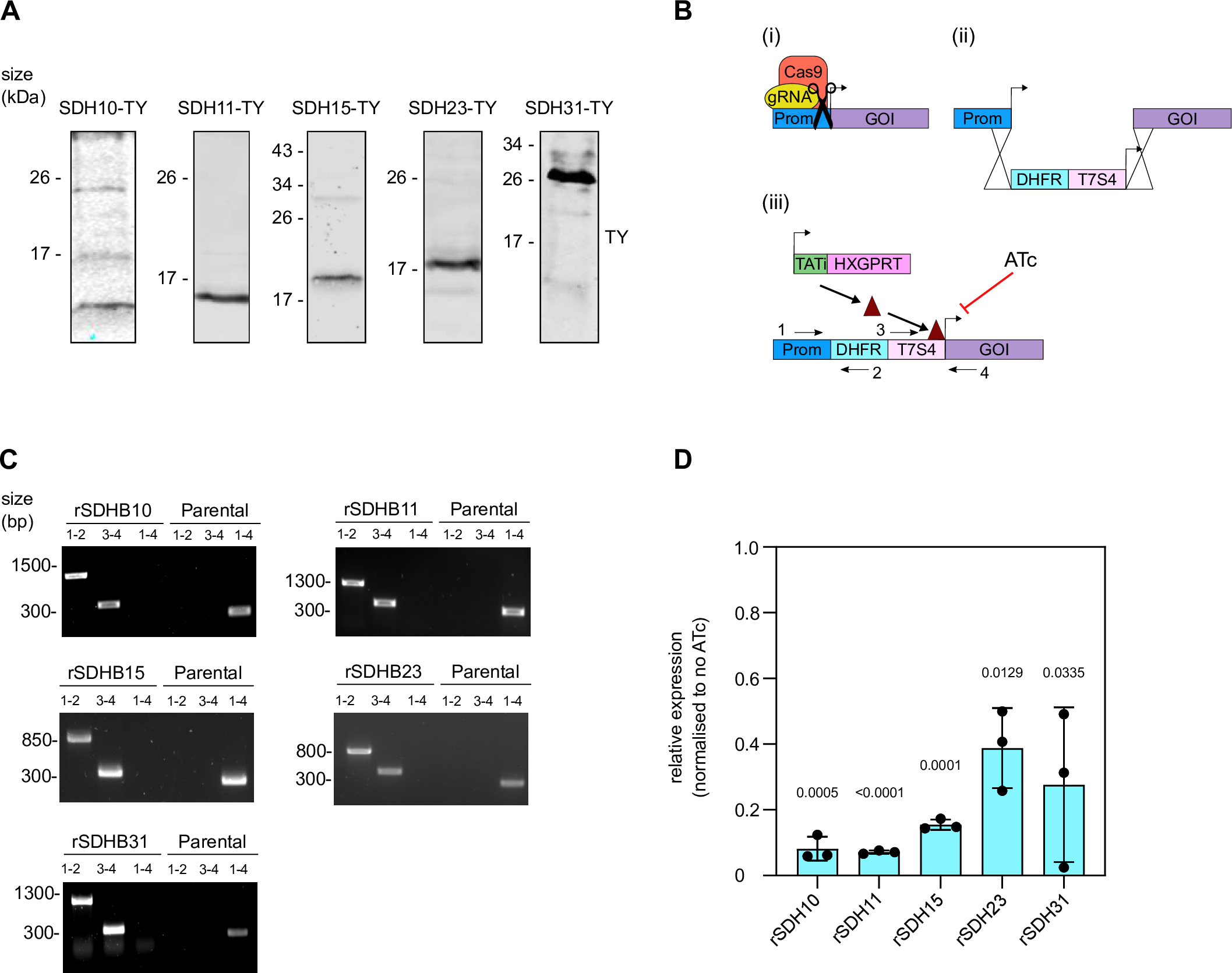

Supplement: S2 Fig — (A) Immunoblot analysis of whole cell lysate extracted from cells expressing SDH10, SDH11, SDH15, SDH23 or SDH31-Ty performed with anti-Ty antibodies. (B) Schematic of the promoter replacement strategy allowing knock-down of a gene of interest (GOI) with the addition of anhydrotetracycline (ATc). (i) CRISPR/CAS9 guided cut at the predicted promoter/ATG boundary, (ii) a repair cassette containing the ATc repressible promoter (T7S4), the dihydrofolate reductase (DHFR) selection marker, and homology to the promoter/ATG boundary, is inserted between the promoter and GOI during cut-repair guided by the homology sequences, (iii) GOI, under the control of the ATc repressible promoter, is down regulated when ATc is added. The black arrows represent the primers used to confirm integration via PCR (primers in S1 Table). (C) PCR analysis of rSDHB10,11,15,23 or 31, using the primers in B, to confirm the integration of the DHFR and repressible promoter. (D) qRT-PCR analysis of relative transcript levels of SDH10,11,15,23 or 31 in the respective promoter replacement lines after growth in ATc for two days, No ATc was set to one and plus ATc conditions were compared by a one-sample t-test. Bars represent the mean ± S.D. (n = 3). (TIF) [file ppat.1011867.s002.tif]

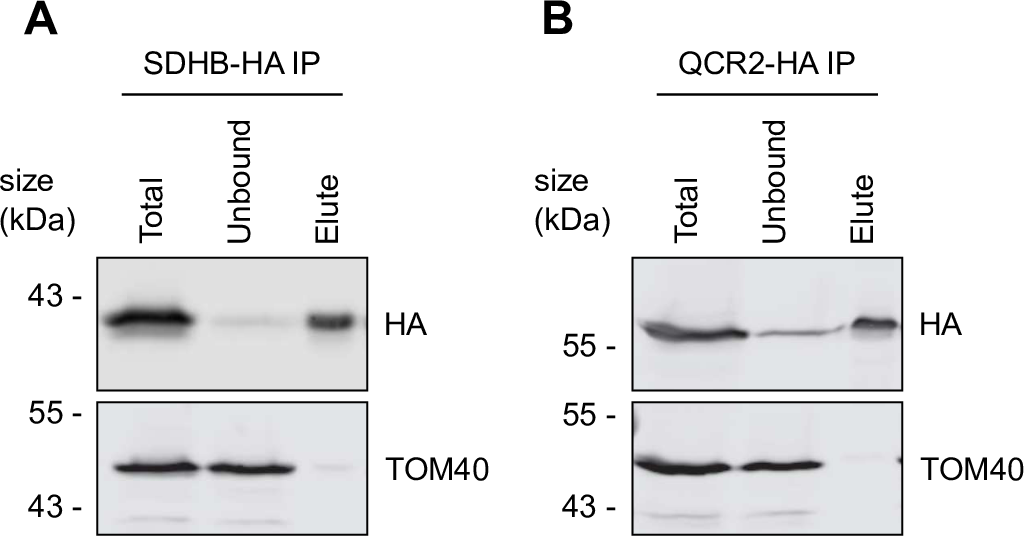

Supplement: S3 Fig — Immunoblot analysis of whole cell lysate extracted from SDHB-HA (A) or QCR2-HA (B) and immunoprecipitated with anti-HA beads, to produce total lysate, unbound and elute fractions. Samples were separated by SDS-PAGE, blotted, and detected using anti-HA antibody to label immunoprecipitated proteins, and anti-TOM40 as an unrelated mitochondrial protein control. (TIF) [file ppat.1011867.s003.tif]

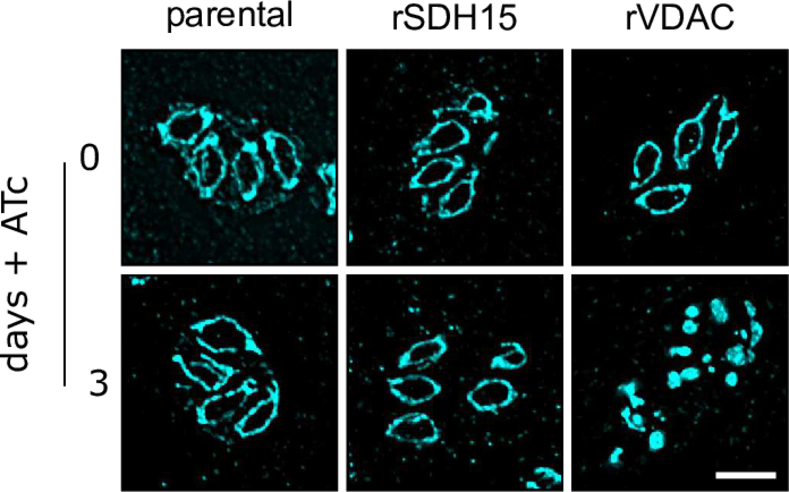

Supplement: S4 Fig — Mitochondrial morphology is visualised with antibodies against the mitochondrial marker protein TOM40. Scale bar is 5 μM. (TIF) [file ppat.1011867.s004.tif]

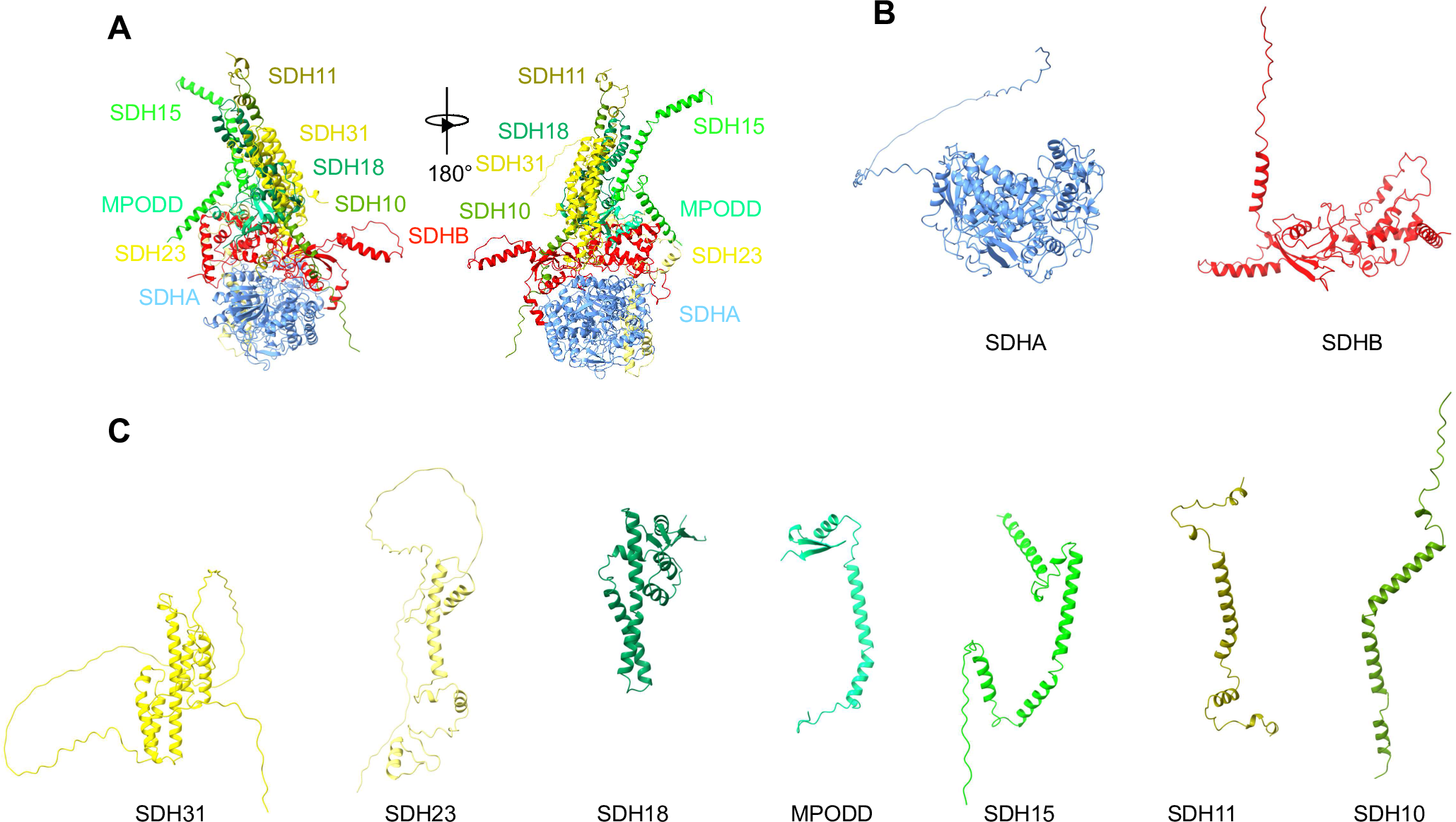

Supplement: S5 Fig — (A) ribbon homology models of Toxoplasma SDH, front and back view, generated using alphafold and matchmaker. Individual subunits colour coded. (B) Homology model of subunits SDHA and SDHB (C) Homology models of the novel SDH subunits. (TIF) [file ppat.1011867.s005.tif]

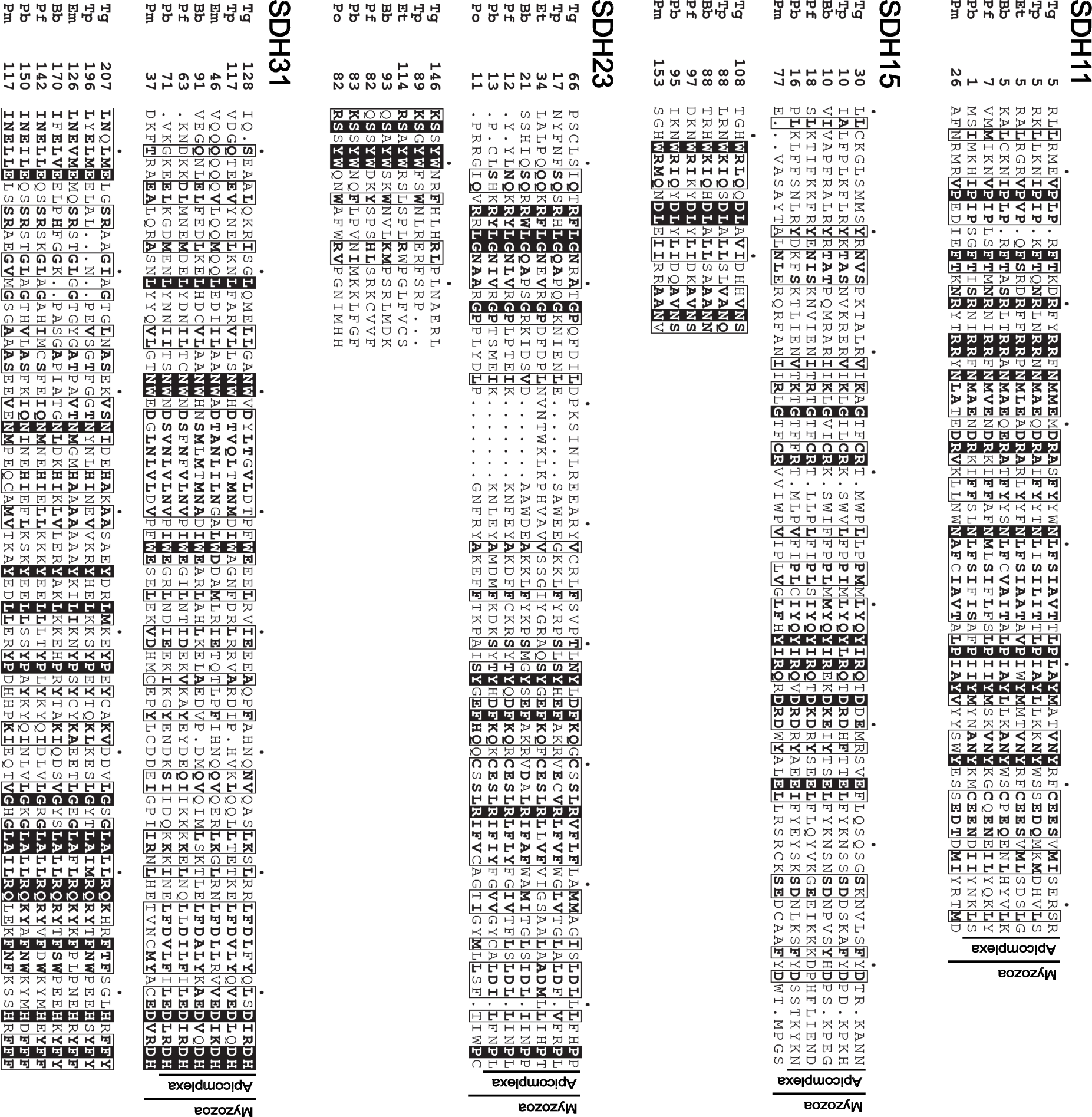

Supplement: S7 Fig — Tg Toxoplasma gondii; Tp, Theileria parva; Et, Eimeria tenella; Bb, Babesia bovis; PF, Plasmodium falciparum; Pb, Plamodium baeghei; Pm, Perkinsus marinu; Po, Perkinsus olseni. (TIF) [file ppat.1011867.s007.tif]
