# Supplementary material for: Functional and biochemical characterization of the Toxoplasma gondii succinate dehydrogenase complex
Source: PLoS Pathog. 2023 Dec 11;19(12):e1011867. doi: 10.1371/journal.ppat.1011867 (PMC10735183; doi:10.1371/journal.ppat.1011867)
Supplement: S6 Fig — Gg, Gallus gallus; Bt, Bos taurus; Hs, Homo sapiens; Dm, Drosophila melanogaster; Bs, Bradyrhizobium species; Bj, Bradyrhizobium japonicum; Ra, Reclinomonas americana; Rp, Rickettsia prowazekii; As, Ascaris suum; Ps, Paracoccus species; Pd, Paracoccus denitrificans; Rr, Rhodospirillum rubrum; Ec, Escherichia coli; Sc, Saccharomyces cerevisiae; Pp, Porphyra purpurea. (PDF) [file ppat.1011867.s006.pdf]

```
      1        10        20        30        40        50            60           70
MPODD MGKLVVPSDISLLEEKQTVGRRRLSVLERLGLMTMPPMIHWNYTKNDK.HDMRQ.....VLQRQYDLSCSDP....ATDI
As    .................MSLLPYNATLCRVLR.HNVKFIRSV..................QTSAARVSAEKTPIQVWGWD
Pp    ................................................................................
Sc    .....MKATIQRV..TSVFGVPRASV..FVPR.ISTPFILHNYISNGR.MDLFSKEFHNGRVSKSDLWSS......NKEE
Rr    ................................................................................
Ps    ................................................................................
Bs    ................................................................................
Ec    ............................................................................MSKA
Dm    .................MYALSS.............SLIRSPALRQGLQMAAAS.....RQVSMKVVSVAET...QKDES
Gg    ..................................................................MATTAK...EEMAR
Bt    ..................................................................LGTTAK...EEMER
Hs    ...................................................MAA.....LLLSAVPLGTTAK...EEMER
Ra    ................................................................................
Rp    ..............................................................................MT

                80                         90           100       110        120
MPODD VVRRQESIRKRVVAHNGVW................AGVAVST....LVGHYSLRRYDYKTKLILLPFIAY....GGSWLG
As    YLMRQRALKRPIAPHLTIYKPQ..MTWMVSGLHRVTGCAMAGTLLIGGVGFSVLPLDF..TTFVEFI...RGLGIPWVIL
Pp    ....MYNINRPISPHLTIYNTQ..KSSLFSIWHRISGVAMFTLIASPPLFLKLATFSYKSFNILDLM.....LNNSSLIL
Sc    ELLVSQRKKRPISPHLTVYEPE..MSWYLSSLHRISGVLLALGFYAFTITLGMTTIMGMDTTFQDLNKWYH.EKMPKWSQ
Rr    ....MKTHPRPISPHLQVYKLHTKVTSMLSITFRVFGAALMVGGTFFFLLWIVAAGSG..EQAFTSVQGF....YGSPFG
Ps    .....MSHRRPKSPNMQNYRPQ..LTSVLSFGHRLSGVAQSLG.ALGLAAWLMAGASG..QDAFGSAQAL....LLSVPG
Bs    ...MTARIERPLSPHMQVYRWT..LTMALSIVHRATGIALYVG.TLLLAWWLIAAASG..PAAYSHVQAF....TGSIIG
Ec    TATQNPARQRPLSPHLSVYRLT..PTMLMSGLHRITGIALYAG.SLLVAWWLIAASIS..PHYFDWVSWA....WGTWIG
Dm    FFEKNERLGRELSPHLTIYQPQ..LTSMLSICHRGTGLALGVGVWGLGLGALISSHDI..SHYVTMV...EGLQLSGATL
Gg    FWEKNTKSSRPLSPHISIYKWS..LPMAMSITHRGTGVALSLGVSLFSLAALLLPEQF..PHYVAVV...KSLSLSPALI
Bt    FWSKNTTLNRPLSPHISIYGWS..LPMAMSICHRGTGIALSAGVSLFGLSALLVPGSF..ESHLEFV...KSLCLGPALI
Hs    FWNKNIGSNRPLSPHITIYSWS..LPMAMSICHRGTGIALSAGVSLFGMSALLLPGNF..ESYLELV...KSLCLGPALI
Ra    .....MNINRPISPHLTIYKLQ..ITNNLSIFHRMTGGALSVVLCFFVIILKMLNFHL..SSYLVYSVIYTVNQFSGFIF
Rp    KIKQEIYNKRPTSPHLTIYKPQ..ISSTLSILHRMTGVALFFVVSILVWWLILSKYDN...NYLQLA........SCCII

                     130       140       150
MPODD RF............LANGLTGRWSEWGRDRALGELPPKAYFEK............................
As    DTFKFIIAFPIAFHTLNGIRFIGFDMAKGTDIPSIYRGAYLVLGLAALISLAVVVYPRWERHKKATLPTNH
Pp    PWFIVIISVIFLYHIINGIRHFLWDSVVNVNTESIIKDSNTLLALVFLIMLFKFIL...............
Sc    WVAKGSAAYLFAFHFGNGIRHLIWDMGYELTNRGVIKTGSIVLAGTLVLGTYLLAQ...............
Rr    YLVLFGLTVVLFYHLCNGIRHMLWDTGFGFDMPTLRKTGAAAVGAAIVLSLLAWTVGLSAVG.........
Ps    RIALFLVTLALFYHLCNGVRHLVWDSGRGFELRAIYAGGWTVVGTSLILTLALWTWGLA............
Bs    RLIVFGYTWALMHHMLSGIRHFVWDLGYGFKANEREALTWGALIGGIVLTVLIWIIAYAIGGGR.......
Ec    RLVLFGYTWALLHHMLGGIRHFIWDVGAVLEKHTATRLATFNLVGSIVLTIAVWVIGYMARGAL.......
Dm    TALKFIIAYPAGYHTANGIRHLLWDTGRFLKIKEVYSTGYAMVATSFVLSAILALL...............
Gg    YSAKFALVFPLSYHTWNGIRHLVWDMGKGFKLSQVEQSGVVVLILTLLSSAAIASE...............
Bt    HTAKFALVFPLMYHTWNGIRHLMWDLGKGLTISQLHQSGVAVLVLTVLSSVGLAAM...............
Hs    HTAKFALVFPLMYHTWNGIRHLMWDLGKGLKIPQLYQSGVVVLVLTVLSSMGLAAM...............
Ra    LMAGFFLILFLFYHLFAGVRHLVWDAGYALEIENVYLSGYIMLGLTFLLTFIVWFIF..............
Rp    KICLVAFSYSWCYHLCNGIRHLFWDIGYGFSIKAVNITGWCVVVCSILLTMLLWV................
```

```
                                                                 1
SDH10 .........................................................MAPSTT................
As    ..........MSLLPYNATLCRVLRHNVKFIRSV.................QTSAARVSAEKTPIQVWGWDYLMRQRAL
Pp    ...........................................................................MYNI
Sc    MKATIQRVTSVFGVPRASV..FVPRISTPFILHNYISNGR.MDLFSKEFHNGRVSKSDLWSS.....NKEEELLVSQRK
Rr    ...........................................................................MKTH
Ps    ............................................................................MSH
Bs    ..........................................................................MTARI
Ec    ...................................................................MSKATATQNPAR
Dm    ..........MYALSS............SLIRSPALRQGLQMAAAS....RQVSMKVVSVAET...QKDESFFEKNERL
Gg    .........................................................MATTAK...EEMARFWEKNTKS
Bt    .........................................................LGTTAK...EEMERFWSKNTTL
Hs    ...........................................MAA....LLLSAVPLGTTAK...EEMERFWNKNIGS
Ra    ............................................................................MNI
Rp    .....................................................................MTKIKQEIYN

        10                  20        30        40        50        60        70
SDH10 PKDASTDAQSWR..........SSSRGIGPRLAATFSWLAL..SSATFAFMTTPRRKEWADICLDYCYSKRAVYAPSVYD
As    KRPIAPHLTIYKPQ..MTWMVSGLHRVTGCAMAGTLLIGGVGFSVLPLDF...TTFVEFI...RGLG.......IPWVIL
Pp    NRPISPHLTIYNTQ..KSSLFSIWHRISGVAMFTLIASPPLFLKLATFSYKS.FNILDLM.....LN.......NSSLIL
Sc    KRPISPHLTVYEPE..MSWYLSSLHRISGVLLALGFYAFTITLGMTTIMGMD.TTFQDLNKWYH.EK.......MPKWSQ
Rr    PRPISPHLQVYKLHTKVTSMLSITFRVFGAALMVGGTFFFLLWIVAAGSG...EQAFTSVQGF...........YGSPFG
Ps    RRPKSPNMQNYRPQ..LTSVLSFGHRLSGVAQSLG.ALGLAAWLMAGASG...QDAFGSAQAL...........LLSVPG
Bs    ERPLSPHMQVYRWT..LTMALSIVHRATGIALYVG.TLLLAWWLIAAASG...PAAYSHVQAF..........TGSIIG
Ec    QRPLSPHLSVYRLT..PTMLMSGLHRITGIALYAG.SLLVAWWLIAASIS...PHYFDWVSWA..........WGTWIG
Dm    GRELSPHLTIYQPQ..LTSMLSICHRGTGLALGVGVWGLGLGALISSHDI...SHYVTMV...EGLQ.......LSGATL
Gg    SRPLSPHISIYKWS..LPMAMSITHRGTGVALSLGVSLFSLAALLLPEQF...PHYVAVV...KSLS.......LSPALI
Bt    NRPLSPHISIYGWS..LPMAMSICHRGTGIALSAGVSLFGLSALLVPGSF...ESHLEFV...KSLC.......LGPALI
Hs    NRPLSPHITIYSWS..LPMAMSICHRGTGIALSAGVSLFGMSALLLPGNF...ESYLELV...KSLC.......LGPALI
Ra    NRPISPHLTIYKLQ..ITNNLSIFHRMTGGALSVVLCFFVIILKMLNFHL...SSYLVYSVIYTVNQ.......FSGFIF
Rp    KRPTSPHLTIYKPQ..ISSTLSILHRMTGVALFFVVSILVWWLILSKYDN....NYLQLA...............SCCII

         80
SDH10 RLFAFLKKVPK............................................................
As    DTFKFIIAFPIAFHTLNGIRFIGFDMAKGTDIPSIYRGAYLVLGLAALISLAVVVYPRWERHKKATLPTNH
Pp    PWFIVIISVIFLYHIINGIRHFLWDSVVNVNTESIIKDSNTLLALVFLIMLFKFIL...............
Sc    WVAKGSAAYLFAFHFGNGIRHLIWDMGYELTNRGVIKTGSIVLAGTLVLGTYLLAQ...............
Rr    YLVLFGLTVVLFYHLCNGIRHMLWDTGFGDMPTLRKTGAAAVGAAIVLSLLAWTVGLSAVG..........
Ps    RIALFLVTLALFYHLCNGVRHLVWDSGRGFELRAIYAGGWTVVGTSLILTLALWTWGLA............
Bs    RLIVFGYTWALMHHMLSGIRHFVWDLGYGFKANEREALTWGALIGGIVLTVLIWIIAYAIGGGR.......
Ec    RLVLFGYTWALLHHMLGGIRHFIWDVGAVLEKHTATRLATFNLVGSIVLTIAVWVIGYMARGAL.......
Dm    TALKFIIAYPAGYHTANGIRHLLWDTGRFLKIKEVYSTGYAMVATSFVLSAILALL...............
Gg    YSAKFALVFPLSYHTWNGIRHLVWDMGKGFKLSQVEQSGVVVLILTLLSSAAIASE...............
Bt    HTAKFALVFPLMYHTWNGIRHLMWDLGKGLTISQLHQSGVAVLVLTVLSSVGLAAM...............
Hs    HTAKFALVFPLMYHTWNGIRHLMWDLGKGLKIPQLYQSGVVVLVLTVLSSMGLAAM...............
Ra    LMAGFFLILFLFYHLFAGVRHLVWDAGYALEIENVYLSGYIMLGLTFLLTFIVWFIF..............
Rp    KICLVAFSYSWCYHLCNGIRHLFWDIGYGFSIKAVNITGWCVVVCSILLTMLLWV................
```

```
                        1        10        20
SDH11  .................MFLSRLLRMEVPLPRFTKDRFYR............RFNMME......................
As     ..........MSLLPYNATLCRVLRHNVKFIRSV..................QTSAARVSAEKTPIQVWGWDYLMRQRAL
Pp     ............................................................................MYNI
Sc     MKATIQRVTSVFGVPRASV..FVPRISTPFILHNYISNGR.MDLFSKEFHNGRVSKSDLWSS......NKEEELLVSQRK
Rr     ............................................................................MKTH
Ps     .............................................................................MSH
Bs     ...........................................................................MTARI
Ec     ....................................................................MSKATATQNPAR
Dm     ..........MYALSS............SLIRSPALRQGLQMAAAS.....RQVSMKVVSVAET...QKDESFFEKNERL
Gg     ..........................................................MATTAK...EEMARFWEKNTKS
Bt     ..........................................................LGTTAK...EEMERFWSKNTTL
Hs     ...........................................MAA.....LLLSAVPLGTTAK...EEMERFWNKNIGS
Ra     .............................................................................MNI
Rp     ......................................................................MTKIKQEIYN

           30          40                                                          50
SDH11  ....MDRASFYYWN..L...FSIA...............................................VTTLPLAYM
As     KRPIAPHLTIYKPQ..MTWMVSGLHRVTGCAMAGTLLIGGVGFSVLPLDF..TTFVEFI...RGLGIPWVILDTFKFIIA
Pp     NRPISPHLTIYNTQ..KSSLFSIWHRISGVAMFTLIASPPLFLKLATFSYKSFNILDLM.....LNNSSLILPWFIVIIS
Sc     KRPISPHLTVYEPE..MSWYLSSLHRISGVLLALGFYAFTITLGMTTIMGMDTTFQDLNKWYH.EKMPKWSQWVAKGSAA
Rr     PRPISPHLQVYKLHTKVTSMLSITFRVFGAALMVGGTFFFLLWIVAAGSG..EQAFTSVQGF....YGSPFGYLVLFGLT
Ps     RRPKSPNMQNYRPQ..LTSVLSFGHRLSGVAQSLG.ALGLAAWLMAGASG..QDAFGSAQAL....LLSVPGRIALFLVT
Bs     ERPLSPHMQVYRWT..LTMALSIVHRATGIALYVG.TLLLAWWLIAAASG..PAAYSHVQAF....TGSIIGRLIVFGYT
Ec     QRPLSPHLSVYRLT..PTMLMSGLHRITGIALYAG.SLLVAWWLIAASIS..PHYFDWVSWA....WGTWIGRLVLFGYT
Dm     GRELSPHLTIYQPQ..LTSMLSICHRGTGLALGVGVWGLGLGALISSHDI..SHYVTMV...EGLQLSGATLTALKFIIA
Gg     SRPLSPHISIYKWS..LPMAMSITHRGTGVALSLGVSLFSLAALLLPEQF..PHYVAVV...KSLSLSPALIYSAKFALV
Bt     NRPLSPHISIYGWS..LPMAMSICHRGTGIALSAGVSLFGLSALLVPGSF..ESHLEFV...KSLCLGPALIHTAKFALV
Hs     NRPLSPHITIYSWS..LPMAMSICHRGTGIALSAGVSLFGMSALLLPGNF..ESYLELV...KSLCLGPALIHTAKFALV
Ra     NRPISPHLTIYKLQ..ITNNLSIFHRMTGGALSVVLCFFVIILKMLNFHL..SSYLVYSVIYTVNQFSGFIFLMAGFFLI
Rp     KRPTSPHLTIYKPQ..ISSTLSILHRMTGVALFFVVSILVWWLILSKYDN...NYLQLA........SCCIIKICLVAFS

             60        70        80        90
SDH11  ATVNYRFCEESVMISERSRGGQLPSDVDRGLFNSEKLTR.................................
As     FPIAFHTLNGIR.........FIGFDMAKGTDIPSIYRGAYLVLGLAALISLAVVVYPRWERHKKATLPTNH
Pp     VIFLYHIINGIR.........HFLWDSVVNVNTESIIKDSNTLLALVFLIMLFKFIL...............
Sc     YLFAFHFGNGIR.........HLIWDMGYELTNRGVIKTGSIVLAGTLVLGTYLLAQ...............
Rr     VVLFYHLCNGIR.........HMLWDTGFGFDMPTLRKTGAAAVGAAIVLSLLAWTVGLSAVG.........
Ps     LALFYHLCNGVR.........HLVWDSGRGFELRAIYAGGWTVVGTSLILTLALWTWGLA............
Bs     WALMHHMLSGIR.........HFVWDLGYGFKANEREALTWGALIGGIVLTVLIWIIAYAIGGGR.......
Ec     WALLHHMLGGIR.........HFIWDVGAVLEKHTATRLATFNLVGSIVLTIAVWVIGYMARGAL.......
Dm     YPAGYHTANGIR.........HLLWDTGRFLKIKEVYSTGYAMVATSFVLSAILALL...............
Gg     FPLSYHTWNGIR.........HLVWDMGKGFKLSQVEQSGVVVLILTLLSSAAIASE...............
Bt     FPLMYHTWNGIR.........HLMWDLGKGLTISQLHQSGVAVLVLTVLSSVGLAAM...............
Hs     FPLMYHTWNGIR.........HLMWDLGKGLKIPQLYQSGVVVLVLTVLSSMGLAAM...............
Ra     LFLFYHLFAGVR.........HLVWDAGYALEIENVYLSGYIMLGLTFLLTFIVWFIF..............
Rp     YSWCYHLCNGIR.........HLFWDIGYGFSIKAVNITGWCVVVCSILLTMLLWV................
```

```
       1        10        20         30            40                    50
SDH15  MSTHSCGNVAG.GAGSSGAAKCNMFRGSARLL.....CKGLSMMSYR......NVS......PKTALRVIKAG...TFCR
As     ...........MSLLPYNATLCRVLRHNVKFIRSV..................QTSAARVSAEKTPIQVWGWDYLMRQRA
Pp     .............................................................................MYN
Sc     MKAT.IQRVTSVFGVPRASV..FVPRISTPFILHNYISNGR.MDLFSKEFHNGRVSKSDLWSS......NKEEELLVSQR
Rr     .............................................................................MKT
Ps     ..............................................................................MS
Bs     ............................................................................MTAR
Ec     .....................................................................MSKATATQNPA
Dm     ...........MYALSS............SLIRSPALRQGLQMAAAS.....RQVSMKVVSVAET...QKDESFFEKNER
Gg     ...........................................................MATTAK...EEMARFWEKNTK
Bt     ...........................................................LGTTAK...EEMERFWSKNTT
Hs     .............................................MAA....LLLSAVPLGTTAK...EEMERFWNKNIG
Ra     ..............................................................................MN
Rp     .......................................................................MTKIKQEIY

       60        70             80                                90
SDH15  TMWPLLPPMMLYQYIR...........QTDDEMRS.....................VEFLQSQ.............SGSK
As     LKRPIAPHLTIYKPQ..MTWMVSGLHRVTGCAMAGTLLIGGVGFSVLPLDF..TTFVEFI...RGLGIPWVILDTFKFII
Pp     INRPISPHLTIYNTQ..KSSLFSIWHRISGVAMFTLIASPPLFLKLATFSYKSFNILDLM.....LNNSSLILPWFIVII
Sc     KKRPISPHLTVYEPE..MSWYLSSLHRISGVLLALGFYAFTITLGMTTIMGMDTTFQDLNKWYH.EKMPKWSQWVAKGSA
Rr     HPRPISPHLQVYKLHTKVTSMLSITFRVFGAALMVGGTFFFLLWIVAAGSG..EQAFTSVQGF....YGSPFGYLVLFGL
Ps     HRRPKSPNMQNYRPQ..LTSVLSFGHRLSGVAQSLG.ALGLAAWLMAGASG..QDAFGSAQAL....LLSVPGRIALFLV
Bs     IERPLSPHMQVYRWT..LTMALSIVHRATGIALYVG.TLLLAWWLIAAASG..PAAYSHVQAF....TGSIIGRLIVFGY
Ec     RQRPLSPHLSVYRLT..PTMLMSGLHRITGIALYAG.SLLVAWWLIAASIS..PHYFDWVSWA....WGTWIGRLVLFGY
Dm     LGRELSPHLTIYQPQ..LTSMLSICHRGTGLALGVGVWGLGLGALISSHDI..SHYVTMV...EGLQLSGATLTALKFII
Gg     SSRPLSPHISIYKWS..LPMAMSITHRGTGVALSLGVSLFSLAALLLPEQF..PHYVAVV...KSLSLSPALIYSAKFAL
Bt     LNRPLSPHISIYGWS..LPMAMSICHRGTGIALSAGVSLFGLSALLVPGSF..ESHLEFV...KSLCLGPALIHTAKFAL
Hs     SNRPLSPHITIYSWS..LPMAMSICHRGTGIALSAGVSLFGMSALLLPGNF..ESYLELV...KSLCLGPALIHTAKFAL
Ra     INRPISPHLTIYKLQ..ITNNLSIFHRMTGGALSVVLCFFVIILKMLNFHL..SSYLVYSVIYTVNQFSGFIFLMAGFFL
Rp     NKRPTSPHLTIYKPQ..ISSTLSILHRMTGVALFFVVSILVWWLILSKYDN...NYLQLA........SCCIIKICLVAF

          100       110       120       130
SDH15  NVLSFYDTRKANNTGHWRLQQDLAVIDHHVNSTQAADVTKDE................................
As     AFPIAFHTLNGIRFIGFDMAKGTDI..........PSIYRGAYLVLGLAALISLAVVVYPRWERHKKATLPTNH
Pp     SVIFLYHIINGIRHFLWDSVVNVNT..........ESIIKDSNTLLALVFLIMLFKFIL...............
Sc     AYLFAFHFGNGIRHLIWDMGYELTN..........RGVIKTGSIVLAGTLVLGTYLLAQ...............
Rr     TVVLFYHLCNGIRHMLWDTGFGFDM..........PTLRKTGAAAVGAAIVLSLLAWTVGLSAVG.........
Ps     TLALFYHLCNGVRHLVWDSGRGFEL..........RAIYAGGWTVVGTSLILTLALWTWGLA............
Bs     TWALMHHMLSGIRHFVWDLGYGFKA..........NEREALTWGALIGGIVLTVLIWIIAYAIGGGR.......
Ec     TWALLHHMLGGIRHFIWDVGAVLEK..........HTATRLATFNLVGSIVLTIAVWVIGYMARGAL.......
Dm     AYPAGYHTANGIRHLLWDTGRFLKI..........KEVYSTGYAMVATSFVLSAILALL...............
Gg     VFPLSYHTWNGIRHLVWDMGKGFKL..........SQVEQSGVVVLILTLLSSAAIASE...............
Bt     VFPLMYHTWNGIRHLMWDLGKGLTI..........SQLHQSGVAVLVLTVLSSVGLAAM...............
Hs     VFPLMYHTWNGIRHLMWDLGKGLKI..........PQLYQSGVVVLVLTVLSSMGLAAM...............
Ra     ILFLFYHLFAGVRHLVWDAGYALEI..........ENVYLSGYIMLGLTFLLTFIVWFIF..............
Rp     SYSWCYHLCNGIRHLFWDIGYGFSI..........KAVNITGWCVVVCSILLTMLLWV................
```

```
                1                  10                   20        30        40
SDH18  ........MRVPGFPS............LTFRADSLL............VQCCIAACVWYGPQDAAAVGLMYWLGRQHG
As     ..........MSLLPYNATLCRVLRHNVKFIRSV.................QTSAARVSAEKTPIQVWGWDYLMRQRAL
Pp     ...........................................................................MYNI
Sc     MKATIQRVTSVFGVPRASV..FVPRISTPFILHNYISNGR.MDLFSKEFHNGRVSKSDLWSS.....NKEEELLVSQRK
Rr     ...........................................................................MKTH
Ps     ............................................................................MSH
Bs     ..........................................................................MTARI
Ec     ...................................................................MSKATATQNPAR
Dm     ..........MYALSS............SLIRSPALRQGLQMAAAS....RQVSMKVVSVAET...QKDESFFEKNERL
Gg     .........................................................MATTAK...EEMARFWEKNTKS
Bt     .........................................................LGTTAK...EEMERFWSKNTTL
Hs     ..........................................MAA.....LLLSAVPLGTTAK...EEMERFWNKNIGS
Ra     ............................................................................MNI
Rp     .....................................................................MTKIKQEIYN

        50        60          70
SDH18  KKVTPPHKLASDGQTALQ...DFLRRKNLT..................................................
As     KRPIAPHLTIYKPQ..MTWMVSGLHRVTGCAMAGTLLIGGVGFSVLPLDF..TTFVEFI...RGLGIPWVILDTFKFIIA
Pp     NRPISPHLTIYNTQ..KSSLFSIWHRISGVAMFTLIASPPLFLKLATFSYKSFNILDLM.....LNNSSLILPWFIVIIS
Sc     KRPISPHLTVYEPE..MSWYLSSLHRISGVLLALGFYAFTITLGMTTIMGMDTTFQDLNKWYH.EKMPKWSQWVAKGSAA
Rr     PRPISPHLQVYKLHTKVTSMLSITFRVFGAALMVGGTFFFLLWIVAAGSG..EQAFTSVQGF....YGSPFGYLVLFGLT
Ps     RRPKSPNMQNYRPQ..LTSVLSFGHRLSGVAQSLG.ALGLAAWLMAGASG..QDAFGSAQAL....LLSVPGRIALFLVT
Bs     ERPLSPHMQVYRWT..LTMALSIVHRATGIALYVG.TLLLAWWLIAAASG..PAAYSHVQAF....TGSIIGRLIVFGYT
Ec     QRPLSPHLSVYRLT..PTMLMSGLHRITGIALYAG.SLLVAWWLIAASIS..PHYFDWVSWA....WGTWIGRLVLFGYT
Dm     GRELSPHLTIYQPQ..LTSMLSICHRGTGLALGVGVWGLGLGALISSHDI..SHYVTMV...EGLQLSGATLTALKFIIA
Gg     SRPLSPHISIYKWS..LPMAMSITHRGTGVALSLGVSLFSLAALLLPEQF..PHYVAVV...KSLSLSPALIYSAKFALV
Bt     NRPLSPHISIYGWS..LPMAMSICHRGTGIALSAGVSLFGLSALLVPGSF..ESHLEFV...KSLCLGPALIHTAKFALV
Hs     NRPLSPHITIYSWS..LPMAMSICHRGTGIALSAGVSLFGMSALLLPGNF..ESYLELV...KSLCLGPALIHTAKFALV
Ra     NRPISPHLTIYKLQ..ITNNLSIFHRMTGGALSVVLCFFVIILKMLNFHL..SSYLVYSVIYTVNQFSGFIFLMAGFFLI
Rp     KRPTSPHLTIYKPQ..ISSTLSILHRMTGVALFFVVSILVWWLILSKYDN...NYLQLA........SCCIIKICLVAFS

                  80                  90
SDH18  ........SQQVTVDWKGANG..........YVNLRS..........................
As     FPIAFHTLNGIRFIGFDMAKGTDIPSIYRGAYLVLGLAALISLAVVVYPRWERHKKATLPTNH
Pp     VIFLYHIINGIRHFLWDSVVNVNTESIIKDSNTLLALVFLIMLFKFIL...............
Sc     YLFAFHFGNGIRHLIWDMGYELTNRGVIKTGSIVLAGTLVLGTYLLAQ...............
Rr     VVLFYHLCNGIRHMLWDTGFGFDMPTLRKTGAAAVGAAIVLSLLAWTVGLSAVG.........
Ps     LALFYHLCNGVRHLVWDSGRGFELRAIYAGGWTVVGTSLILTLALWTWGLA............
Bs     WALMHHMLSGIRHFVWDLGYGFKANEREALTWGALIGGIVLTVLIWIIAYAIGGGR.......
Ec     WALLHHMLGGIRHFIWDVGAVLEKHTATRLATFNLVGSIVLTIAVWVIGYMARGAL.......
Dm     YPAGYHTANGIRHLLWDTGRFLKIKEVYSTGYAMVATSFVLSAILALL...............
Gg     FPLSYHTWNGIRHLVWDMGKGFKLSQVEQSGVVVLILTLLSSAAIASE...............
Bt     FPLMYHTWNGIRHLMWDLGKGLTISQLHQSGVAVLVLTVLSSVGLAAM...............
Hs     FPLMYHTWNGIRHLMWDLGKGLKIPQLYQSGVVVLVLTVLSSMGLAAM...............
Ra     LFLFYHLFAGVRHLVWDAGYALEIENVYLSGYIMLGLTFLLTFIVWFIF..............
Rp     YSWCYHLCNGIRHLFWDIGYGFSIKAVNITGWCVVVCSILLTMLLWV................
```

```
         1         10         20         30         40         50         60         70
SDH23  ..MAFSGLAAMASPPAATRAQL...VSRRFLSGRLACPSAVSLTSGAPFSFAGA....GPFSRCSILT.RNPAASPSCLS
As     ..........MSLLPYNATLCRVLRHNVKFIRS.......V..................QTSAARVSAEKTPIQVWGWDY
Pp     ................................................................................
Sc     MKATIQRVTSVFGVPRASV..FVPRISTPFILH.......NYISNGR.MDLFSKEFHNGRVSKSDLWSS......NKEEE
Rr     ................................................................................
Ps     ................................................................................
Bs     ................................................................................
Ec     ...........................................................................MSKAT
Dm     ..........MYALSS............SLIRS.......PALRQGLQMAAAS.....RQVSMKVVSVAET...QKDESF
Gg     .................................................................MATTAK...EEMARF
Bt     .................................................................LGTTAK...EEMERF
Hs     ..................................................MAA.....LLLSAVPLGTTAK...EEMERF
Ra     ................................................................................
Rp     .............................................................................MTK

                80         90         100                  110        120        130
SDH23  IQTRFLGNRATGPQFDILDPK.......SINLREEAR..YVC..........RLFSVPTLNYLDFKQGCSSLRVFLFLAM
As     LMRQRALKRPIAPHLTIYKPQ..MTWMVSGLHRVTGCAMAGTLLIGGVGFSVLPLDF..TTFVEFI...RGLGIPWVILD
Pp     ...MYNINRPISPHLTIYNTQ..KSSLFSIWHRISGVAMFTLIASPPLFLKLATFSYKSFNILDLM.....LNNSSLILP
Sc     LLVSQRKKRPISPHLTVYEPE..MSWYLSSLHRISGVLLALGFYAFTITLGMTTIMGMDTTFQDLNKWYH.EKMPKWSQW
Rr     ...MKTHPRPISPHLQVYKLHTKVTSMLSITFRVFGAALMVGGTFFFLLWIVAAGSG..EQAFTSVQGF....YGSPFGY
Ps     ....MSHRRPKSPNMQNYRPQ..LTSVLSFGHRLSGVAQSLG.ALGLAAWLMAGASG..QDAFGSAQAL....LLSVPGR
Bs     ..MTARIERPLSPHMQVYRWT..LTMALSIVHRATGIALYVG.TLLLAWWLIAAASG..PAAYSHVQAF....TGSIIGR
Ec     ATQNPARQRPLSPHLSVYRLT..PTMLMSGLHRITGIALYAG.SLLVAWWLIAASIS..PHYFDWVSWA....WGTWIGR
Dm     FEKNERLGRELSPHLTIYQPQ..LTSMLSICHRGTGLALGVGVWGLGLGALISSHDI..SHYVTMV...EGLQLSGATLT
Gg     WEKNTKSSRPLSPHISIYKWS..LPMAMSITHRGTGVALSLGVSLFSLAALLLPEQF..PHYVAVV...KSLSLSPALIY
Bt     WSKNTTLNRPLSPHISIYGWS..LPMAMSICHRGTGIALSAGVSLFGLSALLVPGSF..ESHLEFV...KSLCLGPALIH
Hs     WNKNIGSNRPLSPHITIYSWS..LPMAMSICHRGTGIALSAGVSLFGMSALLLPGNF..ESYLELV...KSLCLGPALIH
Ra     ....MNINRPISPHLTIYKLQ..ITNNLSIFHRMTGGALSVVLCFFVIILKMLNFHL..SSYLVYSVIYTVNQFSGFIFL
Rp     IKQEIYNKRPTSPHLTIYKPQ..ISSTLSILHRMTGVALFFVVSILVWWLILSKYDN...NYLQLA........SCCIIK

           140        150        160        170        180        190        200
SDH23  MAGISLDLL.LFHPPKSSYWNRFHLHRLPLNAERLLFPGKGNVYEYTKNGTKVDPDTGAVATEACASFLKLMYGVSL...
As     TFKFIIAFPIAFH...............TLNGIRFIGFDMAKGTDIP..SIY.RGAYLVLGLAALISLAVVVYPRWERHK
Pp     WFIVIISVIFLYH...............IINGIRHFLWDSVVNVNTE..SII.KDSNTLLALVFLIMLFKFIL.......
Sc     VAKGSAAYLFAFH...............FGNGIRHLIWDMGYELTNR..GVI.KTGSIVLAGTLVLGTYLLAQ.......
Rr     LVLFGLTVVLFYH...............LCNGIRHMLWDTGFGFDMP..TLR.KTGAAAVGAAIVLSLLAWTVGLSAVG.
Ps     IALFLVTLALFYH...............LCNGVRHLVWDSGRGFELR..AIY.AGGWTVVGTSLILTLALWTWGLA....
Bs     LIVFGYTWALMHH...............MLSGIRHFVWDLGYGFKAN..ERE.ALTWGALIGGIVLTVLIWIIAYAIGGG
Ec     LVLFGYTWALLHH...............MLGGIRHFIWDVGAVLEKH..TAT.RLATFNLVGSIVLTIAVWVIGYMARGA
Dm     ALKFIIAYPAGYH...............TANGIRHLLWDTGRFLKIK..EVY.STGYAMVATSFVLSAILALL.......
Gg     SAKFALVFPLSYH...............TWNGIRHLVWDMGKGFKLS..QVE.QSGVVVLILTLLSSAAIASE.......
Bt     TAKFALVFPLMYH...............TWNGIRHLMWDLGKGLTIS..QLH.QSGVAVLVLTVLSSVGLAAM.......
Hs     TAKFALVFPLMYH...............TWNGIRHLMWDLGKGLKIP..QLY.QSGVVVLVLTVLSSMGLAAM.......
Ra     MAGFFLILFLFYH...............LFAGVRHLVWDAGYALEIE..NVY.LSGYIMLGLTFLLTFIVWFIF......
Rp     ICLVAFSYSWCYH...............LCNGIRHLFWDIGYGFSIK..AVN.ITGWCVVVCSILLTMLLWV........

SDH23  ........
As     KATLPTNH
Pp     ........
Sc     ........
Rr     ........
Ps     ........
Bs     R.......
Ec     L.......
Dm     ........
Gg     ........
Bt     ........
Hs     ........
Ra     ........
Rp     ........
```

```
      1        10        20        30        40            50        60        70
SDH31 MPLSLPTARLFARVALTLRGSCCSISCTRYSHLLSTLSVPSCSV.....KNTVGFFPSSRASHFSTASAPATPETVCRPQ
As    ...................................MSLLPYNATLCRVLRHNVKFIRSV....................Q
Pp    ................................................................................
Sc    .................MKA........TIQRVTSVFGVPRASV..FVPRISTPFILHNYISNGR...MDLFSKEFHNGR
Rr    ................................................................................
Ps    ................................................................................
Bs    ................................................................................
Ec    ................................................................................
Dm    ...................................MYALSS............SLIRSPALRQGL..QMAAAS.....RQ
Gg    ................................................................................
Bt    ................................................................................
Hs    ......................................................................MAA.....LL
Ra    ................................................................................
Rp    ................................................................................

         80        90       100       110       120        130       140
SDH31 VSASQSFSSAAPAAAPKSRETGCTAHSETKTNGVARAEDVAAHDFPQLLH.....RE..IQSEAALQKRISGLQMELLGA
As    TSAARVSAEKTPIQ................VWGWDYLMRQRALKRPIAPHLTIYKPQ..MTWMVSGLHRVTGCAMAGTLL
Pp    .......................................MYNINRPISPHLTIYNTQ..KSSLFSIWHRISGVAMFTLIA
Sc    VSKSDLWSS......................NKEEELLVSQRKKRPISPHLTVYEPE..MSWYLSSLHRISGVLLALGFY
Rr    .......................................MKTHPRPISPHLQVYKLHTKVTSMLSITFRVFGAALMVGGT
Ps    ........................................MSHRRPKSPNMQNYRPQ..LTSVLSFGHRLSGVAQSLG.A
Bs    ......................................MTARIERPLSPHMQVYRWT..LTMALSIVHRATGIALYVG.T
Ec    ...............................MSKATATQNPARQRPLSPHLSVYRLT..PTMLMSGLHRITGIALYAG.S
Dm    VSMKVVSVAET...................QKDESFFEKNERLGRELSPHLTIYQPQ..LTSMLSICHRGTGLALGVGVW
Gg    .....MATTAK...................EEMARFWEKNTKSSRPLSPHISIYKWS..LPMAMSITHRGTGVALSLGVS
Bt    .....LGTTAK...................EEMERFWSKNTTLNRPLSPHISIYGWS..LPMAMSICHRGTGIALSAGVS
Hs    LSAVPLGTTAK...................EEMERFWNKNIGSNRPLSPHITIYSWS..LPMAMSICHRGTGIALSAGVS
Ra    ........................................MNINRPISPHLTIYKLQ..ITNNLSIFHRMTGGALSVVLC
Rp    .................................MTKIKQEIYNKRPTSPHLTIYKPQ..ISSTLSILHRMTGVALFFVVS

         150       160       170       180       190       200       210       220
SDH31 ....NWVDYLTGVL.DTPFWEEELRVIEEEAQPFAHNQN..VQASLKSLRRLFDLFYQLSDIRDHLNQLMELGSRAAGIA
As    IGGVGFS.VLPLDF..TTFVEF........I...RGLGIPWVILDTFKFIIAFPIAFHTLNGIRFIGFDMAKGTDIPSIY
Pp    SPPLFLK.LATFSYKSFNILDL........M.....LNNSSLILPWFIVIISVIFLYHIINGIRHFLWDSVVNVNTESII
Sc    AFTITLG.MTTIMGMDTTFQDL........NKWYH.EKMPKWSQWVAKGSAAYLFAFHFGNGIRHLIWDMGYELTNRGVI
Rr    FFFLLWI.VAAGSG..EQAFTS........VQGF....YGSPFGYLVLFGLTVVLFYHLCNGIRHMLWDTGFGFDMPTLR
Ps    LGLAAWL.MAGASG..QDAFGS........AQAL....LLSVPGRIALFLVTLALFYHLCNGVRHLVWDSGRGFELRAIY
Bs    LLLAWWL.IAAASG..PAAYSH........VQAF....TGSIIGRLIVFGYTWALMHHMLSGIRHFVWDLGYGFKANERE
Ec    LLVAWWL.IAASIS..PHYFDW........VSWA....WGTWIGRLVLFGYTWALLHHMLGGIRHFIWDVGAVLEKHTAT
Dm    GLGLGAL.ISSHDI..SHYVTM........V...EGLQLSGATLTALKFIIAYPAGYHTANGIRHLLWDTGRFLKIKEVY
Gg    LFSLAAL.LLPEQF..PHYVAV........V...KSLSLSPALIYSAKFALVFPLSYHTWNGIRHLVWDMGKGFKLSQVE
Bt    LFGLSAL.LVPGSF..ESHLEF........V...KSLCLGPALIHTAKFALVFPLMYHTWNGIRHLMWDLGKGLTISQLH
Hs    LFGMSAL.LLPGNF..ESYLEL........V...KSLCLGPALIHTAKFALVFPLMYHTWNGIRHLMWDLGKGLKIPQLY
Ra    FFVIILK.MLNFHL..SSYLVY........SVIYTVNQFSGFIFLMAGFFLILFLFYHLFAGVRHLVWDAGYALEIENVY
Rp    ILVWWLI.LSKYDN...NYLQL........A........SCCIIKICLVAFSYSWCYHLCNGIRHLFWDIGYGFSIKAVN

              230       240       250       260       270       280
SDH31 GTGLNAS..EKVSNIDEHAKAASAEYDRLMKEYPEYCAKVDDVLGSGLALLRQKHRFTFSGLHRFFY
As    RGAYLVLGLAALISLAVVV....................YPRWERHKKATLPTNH............
Pp    KDSNTLLALVFLIMLFKFI....................L...........................
Sc    KTGSIVLAGTLVLGTYLLA....................Q...........................
Rr    KTGAAAVGAAIVLSLLAWT....................VGLSAVG.....................
Ps    AGGWTVVGTSLILTLALWT....................WGLA........................
Bs    ALTWGALIGGIVLTVLIWI....................IAYAIGGGR...................
Ec    RLATFNLVGSIVLTIAVWV....................IGYMARGAL...................
Dm    STGYAMVATSFVLSAILAL....................L...........................
Gg    QSGVVVLILTLLSSAAIAS....................E...........................
Bt    QSGVAVLVLTVLSSVGLAA....................M...........................
Hs    QSGVVVLVLTVLSSMGLAA....................M...........................
Ra    LSGYIMLGLTFLLTFIVWF....................IF..........................
Rp    ITGWCVVVCSILLTMLLWV................................................
```

```
                    1         10
MPODD  .............MGKLVVPSDISLLEEKQ..................................................
Dm     .......MSLSLLLRGAVRCNAANLVKSARITPLKSYSTLVANVQRKAVVQPLAVAKIVAP..VVRE......ISVSAPR
As     ..............MSLIRCTTSKALKFRQLLKM............A................ARTS......VTTPVSR
Gg     MRSRRIAQRRTDGEMLSLSCGLDNAIEDRALLRG.......TLLHRSAVLTA.AADRSAP..ARQS.......HG.GAPQ
Hs     .........MAVLWRLSAVCG...ALGGRALLLR.......TPVVRPAHISAFLQDRPIPEWCGVQH......IH.LSPS
Bt     ..........MALWRLSVLCG...AREGRALFLR.......TPVVRPALVSAFLQDRPAQGWCGTQH......IH.LSPS
Sc     ................MMLPRSMKFMTGRRIFHTATVRAFQ.STAKKSLTIPFLPVLPQKPGGVRGTPNDAYVPP.....
Ra     ..........................................................................MTEKLL
Ec     ............................................................................MVSN
Pp     ....................................................................MY..KTLLAQVF
Rp     ...................................................................MIYDFKAEIIKAK
Pd     .....................................................................MRYITPRKAAE
Bj     .............................................................MSATDTPKRSMRTPLGRVR
Rr     .....................................................................MSLRSPLGRAR

             20        30        40             50        60        70        80
MPODD  .....TVGRRRLSVLERLGLMTMPPMIHWNY....TKNDKHDMRQVLQRQYDLSCSDPATDIVVRRQESIRKRVVAHNGV
Dm     MASAGSSHTLLWTV.ERIVSAGLLAVI.PAAFIA.....................PSQVLDAL....MAISVVIHTHWGV
As     EPFSIEDHSLHFKI.ERYWAAGMIPLI.PTAYFI.....................HTPAMDAV....LTVAIVLHVHWGI
Gg     GHGSSKAASLHWTS.ERAVSALLLGLL.PAAYLY.....................PGPAVDYS....LAAALTLHGHWGL
Hs     HHSGSKAASLHWTS.ERVVSFLLLGLL.PAAYLN.....................PCSAMDYS....LAAALTLHGHWGL
Bt     HHSGSKAASLHWTG.ERVVSVLLLGLI.PAAYLN.....................PCSAMDYS....LAATLTLHSHWGI
Sc     .PENKLEGSYHWYM.EKIFALSVVPLATTAM....LTTGPLSTA......ADSF.............FSVMLLGYCYMEF
Ra     HFIRTKSGSMHWWL.QRFLAILLAPIILYLLFDVAIYIGQQSDPTVMMFLNRIFNHNSIFIFI....TSVILIWHVRGGM
Ec     ASALGRNGVHDFIL.VRATAIVLTLYIIYMVGFFAT.SGELTYEVWIGFFASAF..TKVFTLL....ALFSILIHAWIGM
Pp     FHSIAK................KKLYFFWLPRLF.......S...LLLVPGFLF..DIEILFL....FHPIILLHASLGL
Rp     NSSFSKSGSHHWLL.QRVTGVILALCSFWLIYFMFT.NKNNDINIIMWEFKKPF..NIVILLI....TVTISLYHSVLGM
Pd     GLGSAHEGTQHHWAM.TVSAVALTVLT.PLFMIVVARAIGLSQEQLLAYFGRPF..PALITAL....FVIVGMVHFIKGT
Bj     NLGAAHSGTSDFWRQ.RITGVAMVLMMIP.VIVIIVMLFGRNQAFVAQTIGSLP..IAIIMLL....FIFASAWHMKIGM
Rr     R.GFRQGGAANHWMAERLPAIALVPLALWFVFVAIISNLGASYAQIQAFMAVPL..NATLMLL....TVFCAFFHGALGL

       90        100                 110       120            130       140       150
MPODD  WAGVAVSTLVGHY........SLR.RYDYKTKLILLP.FIAYGG....SWLGRFLANGLTGRWSEWGRDRALGELPPKAY
Dm     ......EAMVVDYMRPSVVGNVLPKVAHIALIIISV...ATLGGLFYFIQNDVGLANGIKRFWAIKGKDAEKA.......
As     ......AGVVSDYARPFVIGDTLARVARASVYIITV...ILLASLLHFNNSDVGLTKAFEMVWSL...............
Gg     ......GQVITDY....VHGDTPIKVANTGLYVLSA...ITFTGLCYFNYYDVGICKAVAMLWSI...............
Hs     ......GQVVTDY....VHGDALQKAAKAGLLALSA...LTFAGLCYFNYHDVGICKAVAMLWKL...............
Bt     ......GQVVTDY....VHGDAVQKAAKTGLLVLSA...FTFAGLCYFNYHDVGICKAVAMLWKL...............
Sc     ......NSCITDY....ISERVYGVWHKYAMYMLGLGSAVSLFGIYKLETENDGVVGLVKSLWDSSEKDNSQKIEAKK..
Ra     ......EVIIEDY....VHGEKTRIVSIFLIRVIAIE.......IMEYLYKCSIIF........................
Ec     ......WQVLTDY....VKPLALRLMLQLVIV.........VALVVYVIYGFVVVWGV......................
Pp     ......SVIIEDY....IHIETIKFQYLSLIKLLLVL..LINLNILYLL...............................
Rp     ......RVVIEDY....INCHKLRNTLIIIVKLFCIL..TIVSFVVAIFYSG............................
Pd     ......RIMIDDY....FQGGTRKAAIIFSV.IFGWA..VIAAAVYALARMGLGAIVVL.....................
Bj     ......QVVIEDY....VHNEKLKLTAIMLNNFFSIA..VALASTYAILKLSSGV.........................
Rr     ......IVIIEDY....VQNHAVKNALVFGTKLYALF..GAVLAAVSILKLTFGG.........................

MPODD  FEK
Dm     ...
As     ...
Gg     ...
Hs     ...
Bt     ...
Sc     ...
Ra     ...
Ec     ...
Pp     ...
Rp     ...
Pd     ...
Bj     ...
Rr     ...
```

```
                                                                      1       10
SDH10    .............................................................MAPSTTPKDASTDAQSWRS
Dm       .......MSLSLLLRGAVRCNAANLVKSARITPLKSYSTLVANVQRKAVVQPLAVAKIVAP..VVR.........EISVS
As       ..............MSLIRCTTSKALKFRQLLKM............A................ART.........SVTTP
Gg       MRSRRIAQRRTDGEMLSLSCGLDNAIEDRALLRG.......TLLHRSAVLTA.AADRSAP...ARQ.........SHG.G
Hs       .........MAVLWRLSAVCG...ALGGRALLLR.......TPVVRPAHISAFLQDRPIPEWCGVQ.........HIH.L
Bt       ..........MALWRLSVLCG...AREGRALFLR.......TPVVRPALVSAFLQDRPAQGWCGTQ.........HIH.L
Sc       ................MMLPRSMKFMTGRRIFHTATVRAFQS.TAKKSLTIPFLPVLPQKPGGVRGTPNDA...YVPP..
Ra       .............................................................................MTE
Ec       ...............................................................................M
Pp       .......................................................................MY..KTLLA
Rp       ......................................................................MIYDFKAEII
Pd       .....................................................................MR...YITPRK
Bj       .............................................................MSATDTPKRS...MRTPLG
Rr       .....................................................................MS...LRSPLG

        20        30           40        50                   60              70
SDH10    SSRGIGPRLAAT.FSWLALS..SATFAFMTTPRRK...........EWADICLDYCYSKRAVY....A..PSVYDRLFAF
Dm       APRMASAGSSHT.LLWTVERIVSAGLLAVIPAAFI.................................APSQVLDALMAI
As       VSREPFSIEDHS.LHFKIERYWAAGMIPLIPTAYF.................................IHTPAMDAVLTV
Gg       APQGHGSSKAAS.LHWTSERAVSALLLGLLPAAYL.................................YPGPAVDYSLAA
Hs       SPSHHSGSKAAS.LHWTSERVVSFLLLGLLPAAYL.................................NPCSAMDYSLAA
Bt       SPSHHSGSKAAS.LHWTGERVVSVLLLGLIPAAYL.................................NPCSAMDYSLAA
Sc       ....PENKLEGS.YHWYMEKIFALSVVPLATTAMLTTGPLSTAADSFFSVMLLGYCYM......................
Ra       KLLHFIRTKSGS.MHWWLQRFLAILLAPIILYLLF...........DVAIYIGQQSDPTVMMFLNRIFNHNSIFIFITSV
Ec       VSNASALGRNGV.HDFILVRATAIVLTLYIIYMVG...........FFAT.SGELTYEVWIGFFASAF..TKVFTLLALF
Pp       QVFFHSIAK................KKLYFFWLPR...........LF.......S...LLLVPGFLF..DIEILFLFHP
Rp       KAKNSSFSKSGS.HHWLLQRVTGVILALCSFWLIY...........FMFT.NKNNDINIIMWEFKKPF..NIVILLITVT
Pd       AAEGLGSAHEGTQHHWAM.TVSAVALTVLT.PLFM...........IVVARAIGLSQEQLLAYFGRPF..PALITALFVI
Bj       RVRNLGAAHSGTSDFWRQ.RITGVAMVLMMIP.VI...........VIIVMLFGRNQAFVAQTIGSLP..IAIIMLLFIF
Rr       RARR.GFRQGGAANHWMAERLPAIALVPLALWFVF...........VAIISNLGASYAQIQAFMAVPL..NATLMLLTVF

        80
SDH10    LKKVPK..........................................................................
Dm       SVVIHTHWGVEAMVVDYMRPSVVGNVLPKVAHIALIIIS...VATLGGLFYFIQNDVGLANGIKRFWAIKGKDAEKA...
As       AIVLHVHWGIAGVVSDYARPFVIGDTLARVARASVYIIT...VILLASLLHFNNSDVGLTKAFEMVWSL...........
Gg       ALTLHGHWGLGQVITDY....VHGDTPIKVANTGLYVLS...AITFTGLCYFNYYDVGICKAVAMLWSI...........
Hs       ALTLHGHWGLGQVVTDY....VHGDALQKAAKAGLLALS...ALTFAGLCYFNYHDVGICKAVAMLWKL...........
Bt       TLTLHSHWGIGQVVTDY....VHGDAVQKAAKTGLLVLS...AFTFAGLCYFNYHDVGICKAVAMLWKL...........
Sc       ........EFNSCITDY....ISERVYGVWHKYAMYMLGLGSAVSLFGIYKLETENDGVVGLVKSLWDSSEKDNSQKIEA
Ra       ILIWHVRGGMEVIIEDY....VHGEKTRIVSIFLIRVIAIE.......IMEYLYKCSIIF....................
Ec       SILIHAWIGMWQVLTDY....VKPLALRLMLQLVIV.........VALVVYVIYGFVVVWGV..................
Pp       IILLHASLGLSVIIEDY....IHIETIKFQYLSLIKLLLVL..LINLNILYLL...........................
Rp       ISLYHSVLGMRVVIEDY....INCHKLRNTLIIIVKLFCIL..TIVSFVVAIFYSG........................
Pd       VGMVHFIKGTRIMIDDY....FQGGTRKAAIIFSV.IFGWA..VIAAAVYALARMGLGAIVVL.................
Bj       ASAWHMKIGMQVVIEDY....VHNEKLKLTAIMLNNFFSIA..VALASTYAILKLSSGV.....................
Rr       CAFFHGALGLIVIIEDY....VQNHAVKNALVFGTKLYALF..GAVLAAVSILKLTFGG.....................

SDH10    ..
Dm       ..
As       ..
Gg       ..
Hs       ..
Bt       ..
Sc       KK
Ra       ..
Ec       ..
Pp       ..
Rp       ..
Pd       ..
Bj       ..
Rr       ..
```

```
                                             1        10                 20
SDH11  ......................................MFLSRLLRME........VPLPR.FTKDRFY........RRF
Dm     .......MSLSLLLRGAVRCNAANLVKSARITPLKSYSTLVANVQRKAVVQPLAVAKIVAP..VVRE......ISVSAPR
As     ..............MSLIRCTTSKALKFRQLLKM............A................ARTS......VTTPVSR
Gg     MRSRRIAQRRTDGEMLSLSCGLDNAIEDRALLRG.......TLLHRSAVLTA.AADRSAP...ARQS......HG.GAPQ
Hs     .........MAVLWRLSAVCG...ALGGRALLLR.......TPVVRPAHISAFLQDRPIPEWCGVQH......IH.LSPS
Bt     ..........MALWRLSVLCG...AREGRALFLR.......TPVVRPALVSAFLQDRPAQGWCGTQH......IH.LSPS
Sc     ................MMLPRSMKFMTGRRIFHTATVRAFQ.STAKKSLTIPFLPVLPQKPGGVRGTPNDAYVPP.....
Ra     ..........................................................................MTEKLL
Ec     ............................................................................MVSN
Pp     ....................................................................MY..KTLLAQVF
Rp     ...................................................................MIYDFKAEIIKAK
Pd     .....................................................................MRYITPRKAAE
Bj     .............................................................MSATDTPKRSMRTPLGRVR
Rr     .....................................................................MSLRSPLGRAR

          30           40          50                      60                 70
SDH11  NMMEMDRASFYYWN...LFSIAV..TTLPLAYMATV..............NYRFCEE.........SVMISERSRGGQLP
Dm     MASAGSSHTLLWTV.ERIVSAGLLAVI.PAAFIA.....................PSQVLDALMAISVVIHTHWGVEAMV
As     EPFSIEDHSLHFKI.ERYWAAGMIPLI.PTAYFI.....................HTPAMDAVLTVAIVLHVHWGIAGVV
Gg     GHGSSKAASLHWTS.ERAVSALLLGLL.PAAYLY.....................PGPAVDYSLAAALTLHGHWGLGQVI
Hs     HHSGSKAASLHWTS.ERVVSFLLLGLL.PAAYLN.....................PCSAMDYSLAAALTLHGHWGLGQVV
Bt     HHSGSKAASLHWTG.ERVVSVLLLGLI.PAAYLN.....................PCSAMDYSLAATLTLHSHWGIGQVV
Sc     .PENKLEGSYHWYM.EKIFALSVVPLATTAM....LTTGPLSTA......ADSF.........FSVMLLGYCYMEFNSCI
Ra     HFIRTKSGSMHWWL.QRFLAILLAPIILYLLFDVAIYIGQQSDPTVMMFLNRIFNHNSIFIFITSVILIWHVRGMEVII
Ec     ASALGRNGVHDFIL.VRATAIVLTLYIIYMVGFFAT.SGELTYEVWIGFFASAF..TKVFTLLALFSILIHAWIGMWQVL
Pp     FHSIAK................KKLYFFWLPRLF.......S...LLLVPGFLF..DIEILFLFHPIILLHASLGLSVII
Rp     NSSFSKSGSHHWLL.QRVTGVILALCSFWLIYFMFT.NKNNDINIIMWEFKKPF..NIVILLITVTISLYHSVLGMRVVI
Pd     GLGSAHEGTQHHWAM.TVSAVALTVLT.PLFMIVVARAIGLSQEQLLAYFGRPF..PALITALFVIVGMVHFIKGTRIMI
Bj     NLGAAHSGTSDFWRQ.RITGVAMVLMMIP.VIVIIVMLFGRNQAFVAQTIGSLP..IAIIMLLFIFASAWHMKIGMQVVI
Rr     R.GFRQGGAANHWMAERLPAIALVPLALWFVFVAIISNLGASYAQIQAFMAVPL..NATLMLLTVFCAFFHGALGLIVII

        80          90
SDH11  SDVDRGLFNSEKLTR.....................................................
Dm     VDYMRPSVVGNVLPKVAHIALIIISV...ATLGGLFYFIQNDVGLANGIKRFWAIKGKDAEKA.....
As     SDYARPFVIGDTLARVARASVYIITV...ILLASLLHFNNSDVGLTKAFEMVWSL.............
Gg     TDY....VHGDTPIKVANTGLYVLSA...ITFTGLCYFNYYDVGICKAVAMLWSI.............
Hs     TDY....VHGDALQKAAKAGLLALSA...LTFAGLCYFNYHDVGICKAVAMLWKL.............
Bt     TDY....VHGDAVQKAAKTGLLVLSA...FTFAGLCYFNYHDVGICKAVAMLWKL.............
Sc     TDY....ISERVYGVWHKYAMYMLGLGSAVSLFGIYKLETENDGVVGLVKSLWDSSEKDNSQKIEAKK
Ra     EDY....VHGEKTRIVSIFLIRVIAIE.......IMEYLYKCSIIF......................
Ec     TDY....VKPLALRLMLQLVIV.........VALVVYVIYGFVVVWGV....................
Pp     EDY....IHIETIKFQYLSLIKLLLVL..LINLNILYLL.............................
Rp     EDY....INCHKLRNTLIIIVKLFCIL..TIVSFVVAIFYSG..........................
Pd     DDY....FQGGTRKAAIIFSV.IFGWA..VIAAAVYALARMGLGAIVVL...................
Bj     EDY....VHNEKLKLTAIMLNNFFSIA..VALASTYAILKLSSGV.......................
Rr     EDY....VQNHAVKNALVFGTKLYALF..GAVLAAVSILKLTFGG.......................
```

```
       1        10        20        30        40        50        60         70
SDH15  MSTHSCGNVAGGAGSSGAAKCNMFRGSARLLCKGLSMMSYRNVSPKTALRVIKAGTFCRT.MWPLLPPMMLYQYIRQTDD
Dm     ...................MSLSLLLRGAVRCNAANLVKSARITPLKSYSTLVANVQRKAVVQPLAVAKIVAP...VVRE
As     ..........................MSLIRCTTSKALKFRQLLKM............A.................ARTS
Gg     ............MRSRRIAQRRTDGEMLSLSCGLDNAIEDRALLRG.......TLLHRSAVLTA.AADRSAP....ARQS
Hs     .....................MAVLWRLSAVCG...ALGGRALLLR.......TPVVRPAHISAFLQDRPIPEWC.GVQH
Bt     ......................MALWRLSVLCG...AREGRALFLR.......TPVVRPALVSAFLQDRPAQGWC.GTQH
Sc     ............................MMLPRSMKFMTGRRIFHTATVRAFQ.STAKKSLTIPFLPVLPQKPGG.VRGT
Ra     ................................................................................
Ec     ................................................................................
Pp     ................................................................................
Rp     ................................................................................
Pd     ................................................................................
Bj     .........................................................................MS.ATDT
Rr     ................................................................................

       80        90        100       110        120       130
SDH15  EMRSVEFLQSQSGSKNVLSFYDTRKANNTGHWRLQ.QDLAVIDHHVNSTQAADV.......TKD................
Dm     ........IS.....VSAPRMASAGSSHTLLWTV.ERIVSAGLLAVI.PAAFIA.....................PSQVL
As     ........VT.....TPVSREPFSIEDHSLHFKI.ERYWAAGMIPLI.PTAYFI.....................HTPAM
Gg     ........HG......GAPQGHGSSKAASLHWTS.ERAVSALLLGLL.PAAYLY.....................PGPAV
Hs     ........IH......LSPSHHSGSKAASLHWTS.ERVVSFLLLGLL.PAAYLN.....................PCSAM
Bt     ........IH......LSPSHHSGSKAASLHWTG.ERVVSVLLLGLI.PAAYLN.....................PCSAM
Sc     PNDAY..VPP...........PENKLEGSYHWYM.EKIFALSVVPLATTAM....LTTGPLSTA......ADSF......
Ra     .........M.....TEKLLHFIRTKSGSMHWWL.QRFLAILLAPIILYLLFDVAIYIGQQSDPTVMMFLNRIFNHNSIF
Ec     ................MVSNASALGRNGVHDFIL.VRATAIVLTLYIIYMVGFFAT.SGELTYEVWIGFFASAF..TKVF
Pp     .MY....KTL.....LAQVFFHSIAK................KKLYFFWLPRLF.......S...LLLVPGFLF..DIEI
Rp     MIYDF..KAE.....IIKAKNSSFSKSGSHHWLL.QRVTGVILALCSFWLIYFMFT.NKNNDINIIMWEFKKPF..NIVI
Pd     ..MRY..ITP.....RKAAEGLGSAHEGTQHHWAM.TVSAVALTVLT.PLFMIVVARAIGLSQEQLLAYFGRPF..PALI
Bj     PKRSM..RTP.....LGRVRNLGAAHSGTSDFWRQ.RITGVAMVLMMIP.VIVIIVMLFGRNQAFVAQTIGSLP..IAII
Rr     ..MSL..RSP.....LGRARR.GFRQGGAANHWMAERLPAIALVPLALWFVFVAIISNLGASYAQIQAFMAVPL..NATL

SDH15  ..............................E.................................................
Dm     DALMAISVVIHTHWGVEAMVVDYMRPSVVGNVLPKVAHIALIIISV...ATLGGLFYFIQNDVGLANGIKRFWAIKGKDA
As     DAVLTVAIVLHVHWGIAGVVSDYARPFVIGDTLARVARASVYIITV...ILLASLLHFNNSDVGLTKAFEMVWSL.....
Gg     DYSLAAALTLHGHWGLGQVITDY....VHGDTPIKVANTGLYVLSA...ITFTGLCYFNYYDVGICKAVAMLWSI.....
Hs     DYSLAAALTLHGHWGLGQVVTDY....VHGDALQKAAKAGLLALSA...LTFAGLCYFNYHDVGICKAVAMLWKL.....
Bt     DYSLAATLTLHSHWGIGQVVTDY....VHGDAVQKAAKTGLLVLSA...FTFAGLCYFNYHDVGICKAVAMLWKL.....
Sc     ...FSVMLLGYCYMEFNSCITDY....ISERVYGVWHKYAMYMLGLGSAVSLFGIYKLETENDGVVGLVKSLWDSSEKDN
Ra     IFITSVILIWHVRGGMEVIIEDY....VHGEKTRIVSIFLIRVIAIE.......IMEYLYKCSIIF..............
Ec     TLLALFSILIHAWIGMWQVLTDY....VKPLALRLMLQLVIV.........VALVVYVIYGFVVVWGV............
Pp     LFLFHPIILLHASLGLSVIIEDY....IHIETIKFQYLSLIKLLLVL..LINLNILYLL.....................
Rp     LLITVTISLYHSVLGMRVVIEDY....INCHKLRNTLIIIVKLFCIL..TIVSFVVAIFYSG..................
Pd     TALFVIVGMVHFIKGTRIMIDDY....FQGGTRKAAIIFSV.IFGWA..VIAAAVYALARMGLGAIVVL...........
Bj     MLLFIFASAWHMKIGMQVVIEDY....VHNEKLKLTAIMLNNFFSIA..VALASTYAILKLSSGV...............
Rr     MLLTVFCAFFHGALGLIVIIEDY....VQNHAVKNALVFGTKLYALF..GAVLAAVSILKLTFGG...............

SDH15  ........
Dm     EKA.....
As     ........
Gg     ........
Hs     ........
Bt     ........
Sc     SQKIEAKK
Ra     ........
Ec     ........
Pp     ........
Rp     ........
Pd     ........
Bj     ........
Rr     ........
```

```
SDH18   ................................................................................
Dm      .......MSLSLLLRGAVRCNAANLVKSARITPLKSYSTLVANVQRKAVVQPLAVAKIVAP..VVRE......ISVSAPR
As      ..............MSLIRCTTSKALKFRQLLKM............A................ARTS......VTTPVSR
Gg      MRSRRIAQRRTDGEMLSLSCGLDNAIEDRALLRG.......TLLHRSAVLTA.AADRSAP...ARQS......HG.GAPQ
Hs      .........MAVLWRLSAVCG...ALGGRALLLR.......TPVVRPAHISAFLQDRPIPEWCGVQH......IH.LSPS
Bt      ..........MALWRLSVLCG...AREGRALFLR.......TPVVRPALVSAFLQDRPAQGWCGTQH......IH.LSPS
Sc      ................MMLPRSMKFMTGRRIFHTATVRAFQ.STAKKSLTIPFLPVLPQKPGGVRGTPNDAYVPP.....
Ra      ..........................................................................MTEKLL
Ec      ............................................................................MVSN
Pp      ....................................................................MY..KTLLAQVF
Rp      ...................................................................MIYDFKAEIIKAK
Pd      .....................................................................MRYITPRKAAE
Bj      .............................................................MSATDTPKRSMRTPLGRVR
Rr      .....................................................................MSLRSPLGRAR

                                                  1        10        20
SDH18   ..........................................MRVPGFPSLTFRADSLLVQCCIAACVW...........
Dm      MASAGSSHTLLWTV.ERIVSAGLLAVI.PAAFIA.....................PSQVLDALMAISVVIHTHWGVEAMV
As      EPFSIEDHSLHFKI.ERYWAAGMIPLI.PTAYFI.....................HTPAMDAVLTVAIVLHVHWGIAGVV
Gg      GHGSSKAASLHWTS.ERAVSALLLGLL.PAAYLY.....................PGPAVDYSLAAALTLHGHWGLGQVI
Hs      HHSGSKAASLHWTS.ERVVSFLLLGLL.PAAYLN.....................PCSAMDYSLAAALTLHGHWGLGQVV
Bt      HHSGSKAASLHWTG.ERVVSVLLLGLI.PAAYLN.....................PCSAMDYSLAATLTLHSHWGIGQVV
Sc      .PENKLEGSYHWYM.EKIFALSVVPLATTAM....LTTGPLSTA......ADSF.........FSVMLLGYCYMEFNSCI
Ra      HFIRTKSGSMHWWL.QRFLAILLAPIILYLLFDVAIYIGQQSDPTVMMFLNRIFNHNSIFIFITSVILIWHVRGGMEVII
Ec      ASALGRNGVHDFIL.VRATAIVLTLYIIYMVGFFAT.SGELTYEVWIGFFASAF..TKVFTLLALFSILIHAWIGMWQVL
Pp      FHSIAK................KKLYFFWLPRLF.......S...LLLVPGFLF..DIEILFLFHPIILLHASLGLSVII
Rp      NSSFSKSGSHHWLL.QRVTGVILALCSFWLIYFMFT.NKNNDINIIMWEFKKPF..NIVILLITVTISLYHSVLGMRVVI
Pd      GLGSAHEGTQHHWAM.TVSAVALTVLT.PLFMIVVARAIGLSQEQLLAYFGRPF..PALITALFVIVGMVHFIKGTRIMI
Bj      NLGAAHSGTSDFWRQ.RITGVAMVLMMIP.VIVIIVMLFGRNQAFVAQTIGSLP..IAIIMLLFIFASAWHMKIGMQVVI
Rr      R.GFRQGGAANHWMAERLPAIALVPLALWFVFVAIISNLGASYAQIQAFMAVPL..NATLMLLTVFCAFFHGALGLIVII

                                                30        40          50        60
SDH18   .....................................Y..GPQDAAAVGLMYWLGRQHG..KKVTPPHKLASDGQTALQD
Dm      VDYMRPSVVGNVLPKVAHIALIIISV...ATLGGLFYFIQNDVGLANGIKRFWAIKGKDAEKA.................
As      SDYARPFVIGDTLARVARASVYIITV...ILLASLLHFNNSDVGLTKAFEMVWSL.........................
Gg      TDY....VHGDTPIKVANTGLYVLSA...ITFTGLCYFNYYDVGICKAVAMLWSI.........................
Hs      TDY....VHGDALQKAAKAGLLALSA...LTFAGLCYFNYHDVGICKAVAMLWKL.........................
Bt      TDY....VHGDAVQKAAKTGLLVLSA...FTFAGLCYFNYHDVGICKAVAMLWKL.........................
Sc      TDY....ISERVYGVWHKYAMYMLGLGSAVSLFGIYKLETENDGVVGLVKSLWDSSEKDNSQKIEAKK............
Ra      EDY....VHGEKTRIVSIFLIRVIAIE.......IMEYLYKCSIIF..................................
Ec      TDY....VKPLALRLMLQLVIV.........VALVVYVIYGFVVVWGV................................
Pp      EDY....IHIETIKFQYLSLIKLLLVL..LINLNILYLL.........................................
Rp      EDY....INCHKLRNTLIIIVKLFCIL..TIVSFVVAIFYSG......................................
Pd      DDY....FQGGTRKAAIIFSV.IFGWA..VIAAAVYALARMGLGAIVVL...............................
Bj      EDY....VHNEKLKLTAIMLNNFFSIA..VALASTYAILKLSSGV...................................
Rr      EDY....VQNHAVKNALVFGTKLYALF..GAVLAAVSILKLTFGG...................................

          70        80        90
SDH18   FLRRKNLTSQQVTVDWKGANGYVNLRS
Dm      ...........................
As      ...........................
Gg      ...........................
Hs      ...........................
Bt      ...........................
Sc      ...........................
Ra      ...........................
Ec      ...........................
Pp      ...........................
Rp      ...........................
Pd      ...........................
Bj      ...........................
Rr      ...........................
```

```
         1        10        20        30        40        50            60
SDH23    MAFSGLAAMASPPAATRAQLVSRRFLSGRLACPSAVSLTSGAPFSFAGAGPFS....RCSILT...RNPAASPSCL....
Dm       ...............MS....LSLLLRGAVRCNAANLVKSARITPLKSYSTLVANVQRKAVVQPLAVAKIVAP..VVRE.
As       ..........................MSLIRCTTSKALKFRQLLKM............A................ARTS.
Gg       ........MRSRRIAQR....RTDGEMLSLSCGLDNAIEDRALLRG.......TLLHRSAVLTA.AADRSAP...ARQS.
Hs       .....................MAVLWRLSAVCG...ALGGRALLLR.......TPVVRPAHISAFLQDRPIPEWCGVQH.
Bt       ......................MALWRLSVLCG...AREGRALFLR.......TPVVRPALVSAFLQDRPAQGWCGTQH.
Sc       ............................MMLPRSMKFMTGRRIFHTATVRAFQ.STAKKSLTIPFLPVLPQKPGGVRGTP
Ra       ................................................................................
Ec       ................................................................................
Pp       ................................................................................
Rp       ...............................................................................M
Pd       ................................................................................
Bj       .........................................................................MSATDTP
Rr       ................................................................................

               70        80        90        100       110       120       130       140
SDH23    .......SIQTRFLGNRATGPQFDILDPKSINLREEARYVCRLFSVPTLNYLDFKQGCSSLRVFLFLAMMAGISLDLLLF
Dm       .....ISVSAPRMASAGSSHTLLWTV..............ERIVSAGLLAVI........PAAFIA..............
As       .....VTTPVSREPFSIEDHSLHFKI..............ERYWAAGMIPLI........PTAYFI..............
Gg       .....HG.GAPQGHGSSKAASLHWTS..............ERAVSALLLGLL........PAAYLY..............
Hs       .....IH.LSPSHHSGSKAASLHWTS..............ERVVSFLLLGLL........PAAYLN..............
Bt       .....IH.LSPSHHSGSKAASLHWTG..............ERVVSVLLLGLI........PAAYLN..............
Sc       NDAYVPP......PENKLEGSYHWYM..............EKIFALSVVPLA.......TTAM....LTTGPLSTA....
Ra       ......MTEKLLHFIRTKSGSMHWWL..............QRFLAILLAPII.......LYLLFDVAIYIGQQSDPTVMM
Ec       ........MVSNASALGRNGVHDFIL..............VRATAIVLTLYI.......IYMVGFFAT.SGELTYEVWIG
Pp       MY..KTLLAQVFFHSIAK.............................KKLYF.......FWLPRLF.......S...LLL
Rp       IYDFKAEIIKAKNSSFSKSGSHHWLL..............QRVTGVILALCS.......FWLIYFMFT.NKNNDINIIMW
Pd       .MRYITPRKAAEGLGSAHEGTQHHWAM..............TVSAVALTVLT........PLFMIVVARAIGLSQEQLLA
Bj       KRSMRTPLGRVRNLGAAHSGTSDFWRQ..............RITGVAMVLMM.......IP.VIVIIVMLFGRNQAFVAQ
Rr       .MSLRSPLGRARR.GFRQGGAANHWMA.............ERLPAIALVPLA.......LWFVFVAIISNLGASYAQIQA

             150       160          170        180        190       200
SDH23    HPPKSSYWNRFHLHRLPLNAERLLFP...GK.GNVYEYTKNGTKVDPDTGAVATEACASFLKLMYGVS............
Dm       .......PSQV...LDALMAISVVIHTHWGVEAMVVDYMRPS..VVGN....VLPKVAHIALIIISV...ATLGGLFYFI
As       .......HTPA...MDAVLTVAIVLHVHWGIAGVVSDYARPF..VIGD....TLARVARASVYIITV...ILLASLLHFN
Gg       .......PGPA...VDYSLAAALTLHGHWGLGQVITDY......VHGD....TPIKVANTGLYVLSA...ITFTGLCYFN
Hs       .......PCSA...MDYSLAAALTLHGHWGLGQVVTDY......VHGD....ALQKAAKAGLLALSA...LTFAGLCYFN
Bt       .......PCSA...MDYSLAATLTLHSHWGIGQVVTDY......VHGD....AVQKAAKTGLLVLSA...FTFAGLCYFN
Sc       ..ADSF............FSVMLLGYCYMEFNSCITDY......ISER....VYGVWHKYAMYMLGLGSAVSLFGIYKLE
Ra       FLNRIFNHNSI...FIFITSVILIWHVRGGMEVIIEDY......VHGE....KTRIVSIFLIRVIAIE.......IMEYL
Ec       FFASAF..TKV...FTLLALFSILIHAWIGMWQVLTDY......VKPL....ALRLMLQLVIV.........VALVVYVI
Pp       VPGFLF..DIE...ILFLFHPIILLHASLGLSVIIEDY......IHIE....TIKFQYLSLIKLLLVL..LINLNILYLL
Rp       EFKKPF..NIV...ILLITVTISLYHSVLGMRVVIEDY......INCH....KLRNTLIIIVKLFCIL..TIVSFVVAIF
Pd       YFGRPF..PAL...ITALFVIVGMVHFIKGTRIMIDDY......FQGG....TRKAAIIFSV.IFGWA..VIAAAVYALA
Bj       TIGSLP..IAI...IMLLFIFASAWHMKIGMQVVIEDY......VHNE....KLKLTAIMLNNFFSIA..VALASTYAIL
Rr       FMAVPL..NAT...LMLLTVFCAFFHGALGLIVIIEDY......VQNH....AVKNALVFGTKLYALF..GAVLAAVSIL

SDH23    .......L.....................
Dm       QNDVGLANGIKRFWAIKGKDAEKA.....
As       NSDVGLTKAFEMVWSL.............
Gg       YYDVGICKAVAMLWSI.............
Hs       YHDVGICKAVAMLWKL.............
Bt       YHDVGICKAVAMLWKL.............
Sc       TENDGVVGLVKSLWDSSEKDNSQKIEAKK
Ra       YKCSIIF......................
Ec       YGFVVVWGV....................
Pp       .............................
Rp       YSG..........................
Pd       RMGLGAIVVL...................
Bj       KLSSGV.......................
Rr       KLTFGG.......................
```

```
      1        10        20        30        40        50        60        70        80
SDH31 MPLSLPTARLFARVALTLRGSCCSISCTRYSHLLSTLSVPSCSVKNTVGFFPSSRASHFSTASAPATPETVCRPQVSASQ
Dm    ...........MSLSLLLR..................GAVRCNAANLVKSARITPLKSYSTLVANVQ.......RKAVVQ
As    ..................M..................SLIRCTTSKALKFRQLLKM...................A....
Gg    ....MRSRRIAQRRTDGEM..................LSLSCGLDNAIEDRALLRG.......TLLH.......RSAVLT
Hs    .............MAVLWR..................LSAVCG...ALGGRALLLR.......TPVV.......RPAHIS
Bt    ..............MALWR..................LSVLCG...AREGRALFLR.......TPVV.......RPALVS
Sc    .........................................MMLPRSMKFMTGRRIFHTATVRA..FQSTA...KKSLTI
Ra    ................................................................................
Ec    ................................................................................
Pp    ................................................................................
Rp    ................................................................................
Pd    ................................................................................
Bj    ................................................................................
Rr    ................................................................................

                     90        100       110       120       130       140       150
SDH31 SFSSAAPAA......APKS...RETGCTAHSETKTNGVARAEDVAAHDFPQLLHREIQSEAALQKRISGLQMELLGANWV
Dm    PLAVAKIVAP..VVREIS......VSAPRMASAGSSHT.....................LLWTVERIVSAGLLAVIPAAF
As    ............ARTSVT......TPVSREPFSIEDHS.....................LHFKIERYWAAGMIPLIPTAY
Gg    A.AADRSAP...ARQSHG.......GAPQGHGSSKAAS.....................LHWTSERAVSALLLGLLPAAY
Hs    AFLQDRPIPEWCGVQHIH.......LSPSHHSGSKAAS.....................LHWTSERVVSFLLLGLLPAAY
Bt    AFLQDRPAQGWCGTQHIH.......LSPSHHSGSKAAS.....................LHWTGERVVSVLLLGLIPAAY
Sc    PFLPVLPQKPGGVRGTPNDAYVPP......PENKLEGS.......Y.............HWYM.EKIFALSVVPLATTAM
Ra    .......................MTEKLLHFIRTKSGS.......M.............HWWL.QRFLAILLAPIILYLL
Ec    .........................MVSNASALGRNGV.......H.............DFIL.VRATAIVLTLYIIYMV
Pp    .................MY..KTLLAQVFFHSIAK....................................KKLYFFWLP
Rp    ................MIYDFKAEIIKAKNSSFSKSGS.......H.............HWLL.QRVTGVILALCSFWLI
Pd    ..................MRYITPRKAAEGLGSAHEGT.......Q.............HHWAM.TVSAVALTVLT.PLF
Bj    ..........MSATDTPKRSMRTPLGRVRNLGAAHSGT.......S.............DFWRQ.RITGVAMVLMMIP.V
Rr    ..................MSLRSPLGRARR.GFRQGGA.......A.............NHWMAERLPAIALVPLALWFV

                    160                               170       180       190       200
SDH31 ............DYLTGVLDTPF..................WEEELRVIEEEAQPFAHNQNVQASLKSLRRLFDLFYQLS
Dm    ......................IAPSQVLDALMAISVVIHTHWGVEAMVVDYMRPSVVGNVLPKVAHIALIIISVA....
As    ......................FIHTPAMDAVLTVAIVLHVHWGIAGVVSDYARPFVIGDTLARVARASVYIITVI....
Gg    ......................LYPGPAVDYSLAAALTLHGHWGLGQVITDY....VHGDTPIKVANTGLYVLSAI....
Hs    ......................LNPCSAMDYSLAAALTLHGHWGLGQVVTDY....VHGDALQKAAKAGLLALSAL....
Bt    ......................LNPCSAMDYSLAATLTLHSHWGIGQVVTDY....VHGDAVQKAAKTGLLVLSAF....
Sc    ....LTTGPLSTA......ADSF.........FSVMLLGYCYMEFNSCIT....DYISERVYGVWHKYAMYMLGLGSAV.
Ra    FDVAIYIGQQSDPTVMMFLNRIFNHNSIFIFITSVILIWHVRGGMEVIIE....DYVHGEKTRIVSIFLIRVIAIE....
Ec    GFFAT.SGELTYEVWIGFFASAF..TKVFTLLALFSILIHAWIGMWQVLT....DYVKPLALRLMLQLVIV.........
Pp    RLF.......S...LLLVPGFLF..DIEILFLFHPIILLHASLGLSVIIE....DYIHIETIKFQYLSLIKLLLVL..L.
Rp    YFMFT.NKNNDINIIMWEFKKPF..NIVILLITVTISLYHSVLGMRVVIE....DYINCHKLRNTLIIIVKLFCIL..T.
Pd    MIVVARAIGLSQEQLLAYFGRPF..PALITALFVIVGMVHFIKGTRIMID....DYFQGGTRKAAIIFSV.IFGWA..V.
Bj    IVIIVMLFGRNQAFVAQTIGSLP..IAIIMLLFIFASAWHMKIGMQVVIE....DYVHNEKLKLTAIMLNNFFSIA..V.
Rr    FVAIISNLGASYAQIQAFMAVPL..NATLMLLTVFCAFFHGALGLIVIIE....DYVQNHAVKNALVFGTKLYALF..G.

          210       220       230       240       250       260       270       280
SDH31 DIRDHLNQLMELGSRAAGIAGTGLNASEKVSNIDEHAKAASAEYDRLMKEYPEYCAKVDDVLGSGLALLRQKHRFTFSGL
Dm    .TLGGLFYFIQNDVGLANGIKRFWAIKGKDAEKA..............................................
As    .LLASLLHFNNSDVGLTKAFEMVWSL......................................................
Gg    .TFTGLCYFNYYDVGICKAVAMLWSI......................................................
Hs    .TFAGLCYFNYHDVGICKAVAMLWKL......................................................
Bt    .TFAGLCYFNYHDVGICKAVAMLWKL......................................................
Sc    .SLFGIYKLETENDGVVGLVKSLWDSSEKDNSQKIEAKK.........................................
Ra    .....IMEYLYKCSIIF...............................................................
Ec    ..VALVVYVIYGFVVVWGV.............................................................
Pp    .INLNILYLL......................................................................
Rp    .IVSFVVAIFYSG...................................................................
Pd    .IAAAVYALARMGLGAIVVL............................................................
Bj    .ALASTYAILKLSSGV................................................................
Rr    .AVLAAVSILKLTFGG................................................................

SDH31 HRFFY
Dm    .....
As    .....
Gg    .....
Hs    .....
Bt    .....
Sc    .....
Ra    .....
Ec    .....
Pp    .....
Rp    .....
Pd    .....
Bj    .....
Rr    .....
```
