# Supplementary material for: Functional and biochemical characterization of the Toxoplasma gondii succinate dehydrogenase complex
Source: PLoS Pathog. 2023 Dec 11;19(12):e1011867. doi: 10.1371/journal.ppat.1011867 (PMC10735183; doi:10.1371/journal.ppat.1011867)
Supplement: Attachment — Submitted filename: SilvaRespondToReviewer.docx [file ppat.1011867.s012.docx]

*Dear Michael, Meg, Kasturi and Michael,*

*We thank you and the reviewers for taking the time to revise our manuscript and for the constructive comments that helped us improved it. We addressed almost all the comments with a revision to the manuscript and where possible with new experiments. Below, where the editor and reviewer comments are in black plain text, we explain how we addressed each comment in italic and green text, where underlined text highlights the specific revision performed in response to a comment.*

*Sincerely,*

*Andrew and Lilach*

Dear Dr. Sheiner,

Thank you very much for submitting your manuscript "Functional and biochemical characterisation of the Toxoplasma gondii succinate dehydrogenase complex" for consideration at PLOS Pathogens. As with all papers reviewed by the journal, your manuscript was reviewed by members of the editorial board and by several independent reviewers. The reviewers appreciated the attention to an important topic. Based on the reviews, we are likely to accept this manuscript for publication, providing that you modify the manuscript according to the review recommendations.

As you will see, both reviewers were overall positive about the manuscript. Please pay close attention to the in vitro experiments requested, as they seem reasonable to support the central claims made in the paper. I would also agree with Reviewer #2 that mouse experiments are required to support any claims of an in vivo requirement for something not essential in vitro. Please address this concern either experimentally or by modifying the manuscript.

Please prepare and submit your revised manuscript within 30 days. If you anticipate any delay, please let us know the expected resubmission date by replying to this email.

When you are ready to resubmit, please upload the following:

[1] A letter containing a detailed list of your responses to all review comments, and a description of the changes you have made in the manuscript.

Please note while forming your response, if your article is accepted, you may have the opportunity to make the peer review history publicly available. The record will include editor decision letters (with reviews) and your responses to reviewer comments. If eligible, we will contact you to opt in or out

[2] Two versions of the revised manuscript: one with either highlights or tracked changes denoting where the text has been changed; the other a clean version (uploaded as the manuscript file).

Important additional instructions are given below your reviewer comments.

Thank you again for your submission to our journal. We hope that our editorial process has been constructive so far, and we welcome your feedback at any time. Please don't hesitate to contact us if you have any questions or comments.

Sincerely,

Michael L Reese, PhD

Academic Editor

PLOS Pathogens

Margaret Phillips

Section Editor

PLOS Pathogens

Kasturi Haldar

Editor-in-Chief

PLOS Pathogens

orcid.org/0000-0001-5065-158X

Michael Malim

Editor-in-Chief

PLOS Pathogens

orcid.org/0000-0002-7699-2064

***********************

As you will see, both reviewers were overall positive about the manuscript. Please pay close attention to the in vitro experiments requested, as they seem reasonable to support the central claims made in the paper. I would also agree with Reviewer #2 that mouse experiments are required to support any claims of an in vivo requirement for something not essential in vitro. Please address this concern either experimentally or by modifying the manuscript.

Reviewer Comments (if any, and for reference):

Reviewer's Responses to Questions

**Part I - Summary**
Please use this section to discuss strengths/weaknesses of study, novelty/significance, general execution and scholarship.

Reviewer #1: The authors test the localization, essentiality, and function of several new, parasite-specific proteins identified in a prior complexome profile of ETC complex II in Toxoplasma gondii. Their prior study had identified two of the four canonical members of SDH known in mammals but suggested seven novel subunits in parasites. This study confirms five of the seven proteins as core members of complex II and provides direct evidence that complex II function is critical for parasites.

This strong study is well executed and features good controls. The results and conclusions impactfully advance understanding of the divergent composition of apicomplexan ETC complex II. There are a few inconsistencies and questions noted below, answering which will tighten and strengthen the major conclusions and implications.

Reviewer #2: In the reported work, Marina Silva et al verified that several novel subunits of the purported mitochondrial complex II previously identified in complexomic studies are indeed components of a large mitochondrial complex and are required for succinate-ubiquinone oxidoreductase activity in the parasite, as expected for complex II components. The experiments addressing these points appear to be thorough and the results compelling. Statements by the authors regarding the suitability of SDH/complex II as a drug target in Toxoplasma and other apicomplexans are less convincing since the subunits proved not strictly necessary for growth in vitro. Thus the study should be bolstered by in vivo experiments with knockdown parasite lines.

**Part II – Major Issues: Key Experiments Required for Acceptance**
Please use this section to detail the key new experiments or modifications of existing experiments that should be absolutely required to validate study conclusions.
Generally, there should be no more than 3 such required experiments or major modifications for a "Major Revision" recommendation. If more than 3 experiments are necessary to validate the study conclusions, then you are encouraged to recommend "Reject".

Reviewer #1: 1. Lines 143-146: The gels in Fig. 1C are not convincing that all 4 proteins migrate in the same-sized complex ~500 kDa, since the bands for 10 and 31 appear to be ~700 based on the markers. Since the MW sum of all 9 proposed SDH proteins is ~230 kDa (Table 1), the authors are presumably detecting a higher-order oligomeric complex, but this aspect is not discussed. One explanation for the differing BN-PAGE band sizes could be that these proteins are present in distinct oligomeric states for SDH. Idiosyncrasy and gel-to-gel variability are also normal in BN-PAGE experiments. To better resolve and understand these differences, can the authors run lysates from the four tagged lines on the same BN-PAGE gel (and/or co-load lysates from different tags in the same lane) to directly establish if the four proteins are in dominant complexes of similar or distinct sizes?

*We agree that a run on the same gel provides better support that all proteins migrate similarly, we performed this experiment and added it to the revised figures Fig 1C and S Fig 1D. The lines contain ectopic copies which are expressed at different levels, and this was a challenge for getting clarity of all four signals in one gel exposure. For this reason, Fig 1C shows separate lanes from different exposures, while S Fig 1D showed the full unprocessed gel.*

2. The structural homology modelling in Fig. 5C is interesting and insightful. It would be very helpful if the authors could label the individual parasite proteins modelled in this panel.

*We agree that a colour-coding and labelling improve the clarity of the predicted model, we provide this in the revised Fig 5C and we generated a new S Fig 5 with front and back view and with the predictions of the folding of each subunit.*

3. For Fig. 6D, does the DY-AA mutation destabilize SDH10 and thus result in diminished levels of this protein (that are insufficient to stabilize complex II upon knockdown of endogenous SDH10?) The authors can detect the mutant protein in the mitochondrion but this IFA does not enable evaluation of relative protein levels. Probing for TY in the Fig. 6D blot would be sufficient to assess.

*We agree that analysis of the expression level of SDH10 DY-AA level provides more support to the observation that this mutation results in reduction in complex stability. We performed the western blot again and add it to the revised Fig 6B. To our surprise, not only this protein is stable, but its levels are elevated. We comment about the fact that the protein is stable and report about the increase (line 424). Understanding the mechanism, awaits future work, therefore we do not discuss it at this point hypothesise about it.*

Reviewer #2: An important part of the claimed significance of this work is the potential of the SDH complex to be a drug target in Toxoplasma and other apicomplexan parasites. This should be supported by experiments in animals since the complex was found not to be absolutely required for propagation in vitro.

*We will answer this important point in three ways:*

***In vitro growth defect****: we agree with the reviewer that our data indicates that while the SDH subunits are important for fitness, they may not be absolutely required for propagation in vitro. The caveat here, as highlighted by reviewer 1, is that the knock-down may still have residual protein that results in the growth which we cannot exclude. We now discuss this point in line 319.*

***In vivo growth****: before running our own animal experiments, we consulted with Chris Giuliano from the Lourido group, who recently performed a CRISPR screens to functionally profile Toxoplasma genes during mouse infection (https://www.biorxiv.org/content/10.1101/2023.03.05.531216v1). Since the database is not accessible yet, Chris was kind enough to check about SDH subunit encoding genes for us. Chris found that while there is variability between the genes, most of them are expected to have similar defect in mice compared to cell-culture, namely that knockouts of these genes would all have decreased virulence in mice. While there was some variability that might prompt us to look into individual genes in the future, these observations convinced us that animal experiments may not add more support for the importance of these genes in vivo beyond what has been done in vitro. Because the database is not available yet, and due to PLOS p policy about “data not shown”, we do not discuss this point in the manuscript, but we will opt for this document with answers to reviewer comments to be made available to readers.*

***Discussion of the potential of SDH as a drug target****: we agree that so long as we don’t have data from clean knock-outs it is preliminary to suggest strongly that SDH is a good target for drugs. We thus changed those claims in the abstract (line 25, 52) and introduction (line 87). We do think that the fitness defect observed leaves this notion not without merit, and may at least highlight a utility as target for combination therapy, and we refer to this in a careful way in the discussion (line 500).*

**Part III – Minor Issues: Editorial and Data Presentation Modifications**
Please use this section for editorial suggestions as well as relatively minor modifications of existing data that would enhance clarity.

Reviewer #1: 1. Lines 37-38: should be “starts” and “studies”

*This is now corrected (now line 36-37)*

2. Line 63: should be “have”

*This is now corrected (now line 70)*

3. Lines 63-69: molecular divergence by itself doesn’t seem the most compelling rationale for studying these complexes in Apicomplexa. Rather, the implications and insights of this divergence for understanding pathogen evolution and identifying pathogen-specific metabolic vulnerabilities distinct from the host seem to be the major motivators for defining and understanding these differences from mammals (and yeast)

*We have added lines 73-75 to highlight this.*

4. Lines 74-79: These statements are overly general, since complex II/SDH (Pubmed 25843709 and 22628552) and ATP synthase (Pubmed 25831536) are not essential for blood-stage Plasmodium.

*We amended this sentence to be more detailed (line 83-85)*

5. Does complex II or any of the new membrane-bound subunits bind heme? Does the BN-PAGE band ~500 kDa give a positive “heme stain” by luminol-based chemiluminescence (e.g., Pubmed 12672416)? The role of heme in SDH in mammalian and yeast complex II remains uncertain but appears to contribute to complex stability. Given the paucity of heme-dependent proteins in apicomplexan parasites relative to mammals, understanding of whether T gondii SDH retains or lacks bound heme would further clarify molecular similarity or differences from opisthokonts.

*This is a good question which we were keen to answer too, and we have attempted to perform assays to study it, but these were unsuccessful and will require further optimisation. We hope to be able to follow up with research about the role of heme in the future.*

6. Lines 134-136: Based on current Fig. 1B, it is not obvious that SDHB and TOM40 have resolvably distinct localization within mitochondria. Higher magnification and an intensity vs distance plot along a line orthogonal to the membranes showing discrete intensity maxima would be needed to convincingly make this conclusion.

*We agree that better resolution improved this distinction of localisations, and we now provide data from expansion microscopy in the revised Fig 1B, and we have added SDH15 to the analysis. We moved the data from super resolution microscopy that was in the original figure to the revised S Fig1A, and we also include the intensity vs distant plot performed on the new expansion microcopy data into that revised S Fig C.*

7. Can the authors provide the full mass spec data for pull-downs of SDHA, SDHB, and cyt c1 to better evaluate interaction specificity? Table S2 is presumably an over-simplification of interactors, and pull-down with many more proteins was observed in all cases.

*We have now performed new analysis, with a more advanced mass spec method, which supersedes the original data. We have thus replaced the data in the original paper. Quantitative IP that provides strong evidence of the new subunits in the SDH complex has been added to revised Fig. 1E and S Fig 3, described in the revised text (lines 189-218), and full mas spec data provides in S Table 2.*

8. Lines 190-191: reduction in SDHB-HA levels upon knockdown of other subunits seems to correlate with loss of the ~500 kDa band, which suggests that SDHB stability depends on complex II integrity. The authors may wish to explicitly discuss this connection.

*We added a comment about this observation in lines 250-252.*

9. Line 601: the authors presumably mean “/mM /cm” for the extinction coefficient units.

*This is now corrected (now line 808)*

10. Line 209: the authors describe use of parasite lysates here but the methods (lines 590-601) only mention intact parasites. Can the authors check and clarify what sample they used and how they normalized protein concentrations across samples?

*This has been revised, no in lines 802, 805-806.*

11. Line 852: Does “three independent experiments” mean technical (same samples run three times) or biological (three different samples derived from different parasite knockdown experiments) replicates?

*This were biological, which is now stated in the revised line 1211.*

12. Lines 93 and 243: Apicomplexa like Plasmodium lack canonical multi-subunit complex I but express a single-subunit type II NADH dehydrogenase that is expected to generate reduced ubiquinol which contributes electrons to the ETC (e.g., Pubmed 30964863). The authors’ statements here seem a bit misleading.

*We revised this in lines 101-102 and 313.*

13. Lines 249-250 and 421-423: An alternative explanation is that SDH is essential for Toxoplasma but the knockdowns of SDH subunits were insufficiently stringent to fully ablate SDH activity, resulting in residual parasite growth. More detailed experiments would be needed to differentiate essential versus fitness conferring (but not strictly essential) contributions. The authors may wish to acknowledge this possibility, especially as protein levels were not examined after ATc addition.

*This is a good point and we commented about this in the revised text lines 319-320.*

14. Fig. 4F: The authors should include a “no ATc” or parental control to establish baseline differentiation for comparison to the two experimental knockdowns.

*The no ATC parental control is included in S Table 4.*

15. Opisthokonts appears to be misspelled as “opistokonts” in multiple locations (lines 352,355, 402).

*We changed this in the following lines: 447, 444, 510*

16. Are SDH knockdown parasites (tachyzoites or bradyzoites) more sensitive to atovaquone (additive or synergistic)? Atovaquone monotherapy is associated with partial efficacy, which limits its utility for treating chronic or acute toxoplasmosis. Would combinatorial inhibition of SDH and atovaquone treatment prove beneficial for more stringently killing of parasites and preventing recrudescence? This experiment is not required for the authors’ conclusions but would substantially increase the translational relevance of the present study.

*We agree that this would enhance the contribution of our work to future translational studies, however since the parasites have already a strong fitness cost through the loss of SDH, we failed to be able to evaluate the IC50 with a replication assay.*

Reviewer #2: Lines 74-75: "essential throughout their complicated life cycles" doesn't apply to all apicomplexans, i.e. Cryptosporidium, but also P. berghei (manuscript ref 11).

*We revised the text to be more specific, lines 83-85.*

line 78: "dual target" inhibitors of Plasmodium may have been explored, but the lethality is almost certainly due to inhibition of complex III since SDH is clearly dispensable in the blood stage (similar to the case recently reported for NADH dehydrogenase inhibitors that also inhibit complex III in P. falciparum).

*We removed the discussion of the previously discussed potential SDH inhibition (in response to this comment but also to the above comment about revising our discussion of SDH as a potential drug target), line 87.*

line 93-94 and line 243: "in the absence of mETC complex I, apicomplexan SDH is one of the first points of entry for electrons..." The absence of complex I is irrelevant since there are NADH dehydrogenases, as well as other quinone-dependent dehydrogenases, that all donate electrons at the same entry point as SDH.

*We revised this which in now in lines 101-102 and 313.*

line 360 and Table I: "study by Zwahlen et al; provided validation for the additional two, SDH18 and MPODD" Zwahlen et al is an important reference but is not listed in the reference section.

*The study by Zwahlen et al is still under preparation, we thus cite our communication with Giel Van Dooren and Soraya Zwahlen instead (lines 453, 1282).*

line 366: "substrate" Since there are multiple substrates, specify that it is ubiquinone.

*The was changed, now in line 459.*

line 378: "validated that all five proteins are membrane bound," "Validated" seems to be too strong a conclusion, since some may be bound indirectly via binding to another integral membrane subunit, which is suggested by the subunits predicted to lack TM segments (Table I: SDHB, SDH15, SDH31, MPODD).

*Note that the sentence refers to the five new subunits we studied (which do not include SDHB, SDH31 and MPOD), and that SDH15 is predicted by one algorithm to be membrane bound. Nevertheless, we changed the wording to “strong support” line 486.*

line 386: "demonstrated a dependence of SDH function on this DY motif." Should be "structure or function", i.e., a trivial reason for lack of function is failure to fold correctly. This is similar to the qualifications stated by the authors in the following sentence, but even is a mutated protein is correctly localized, there may be conformational changes that block function.

*The was changed, now in line 494.*

line 421: "it is expected that SDH function will be essential for the parasites" I wouldn't expect it to be essential since there are several other dehydrogenases that donate electrons to ubiquinone (and it isn't essential in Plasmodium).

*The was changed to discuss fitness, now in line 494.*

Fig. 3A: Next to last bar is mislabeled.

*The was corrected in revised Fig 3.*

Fig. 5: suggest moderately enlarging B and C, and color-coding the gray subunits so the individual subunits having membrane segments can be identified and compared to predictions in Table I.

*We colour coded the subunits and provided and provided the predicted fold of each subunit separately in a new S Fig 5*

Table I: One of the TMD predictors gives predictions that mostly differ from the other two and happens to not be peer reviewed. Suggest substituting results from a peer reviewed algorithm. One possibility that combines the predictions of multiple predictors is CCTOP [László Dobson, István Reményi and Gábor E. Tusnády (2015), Nucleic Acids Research, Webserver issue (43) doi: 10.1093/nar/gkv451]

*We added this perdition to the revised Table 1.*
