# Supplementary material for: Functional and biochemical characterization of the Toxoplasma gondii succinate dehydrogenase complex
Source: PLoS Pathog. 2023 Dec 11;19(12):e1011867. doi: 10.1371/journal.ppat.1011867 (PMC10735183; doi:10.1371/journal.ppat.1011867)
Supplement: Attachment — Submitted filename: Respond_to_reviewrs2.docx [file ppat.1011867.s013.docx]

**Dear Michael, Meg, Kasturi and Michael**

**Thank you once again for reviewing our manuscript and providing further feedback. We are thrilled to receive the positive feedback and resubmit our manuscript accordingly. Below is a summary of what we did (in green and bold) in respond to the new comments.**

**Best wishes**

**Andrew and Lilach**

Both reviewers feel that previous concerns were adequately addressed, though there is a suggestion from reviewer #1 to make a minor alteration to the text regarding adding some discussion of data that is otherwise somewhat ignored. I agree that this would likely strengthen the manuscript.

**We have added a comments about this point in the revised manuscript.**

With regard to citing unpublished data -- "Personal communication" citations have fallen out of favor for very good reason. That is what bioRxiv is for. If there is no preprint of unpublished data, it seems inappropriate to cite it. A reader has no way of assessing the relevant cited source for themselves, and at a later date has no way of knowing whether or not (or where) the data was eventually published. I would suggest removing this citation unless such a preprint now exists.

**Unfortunately, our colleague were unable to release a preprint of their work. We have therefore removed the reference to their unpublished data.**

**Part I - Summary**

Reviewer #1: The authors have adequately addressed the prior critiques via responsive modifications. This manuscript will be a strong addition to the apicomplexan literature on ETC function. Two minor suggestions that might be addressed in the final manuscript are listed below.

Reviewer #2: In the reported work, Marina Silva et al verified that several novel subunits of the purported Toxoplasma mitochondrial complex II previously identified in complexomic studies are indeed components of a large mitochondrial complex and are required for succinate-ubiquinone oxidoreductase activity in the parasite, as expected for complex II components. The experiments addressing these points appear to be thorough and the results compelling.

**We’d like to thank both reviewers again for the time taken to provide feedback that improved our manuscript.**

**Part II – Major Issues: Key Experiments Required for Acceptance**
Reviewer #1: None

Reviewer #2: The authors have adequately addressed this reviewer's question regarding claimed potential of complex II to be a drug target in Toxoplasma and other apicomplexan parasites by appropriate modifications of the text and reference to pre-publication in vivo results from another well-regarded Group.

**Part III – Minor Issues: Editorial and Data Presentation Modifications**

Reviewer #1: 1. For the data in Figure 6B that indicate increased episomal SDH10-DY/AA protein levels upon Atc addition, can the authors extend the sentence in lines 354-355 to propose a possible biochemical basis for this increase? It seems odd to note this increase without any interpretation.

**We have added a comment about this observation both, in the relevant context within the result section as suggested by the reviewer, and in the discussion section (lines 403-407).**

2. This reviewer appreciates the authors' dilemma in not being able to cite a formal van Dooren reference (line 371), since their manuscript is still in preparation. The editors will know best what format conforms with journal standards, but it looks like situations like this one require description as a "personal communication" and an accompanying letter from the relevant authors making the communication of unpublished results: <https://journals.plos.org/plospathogens/s/submission-guidelines#:~:text=Do%20not%20cite%20the%20following,in%20a%20publicly%20available%20database>.

Reviewer #2: In this revision the authors have addressed the points previously raised by this reviewer. However, in their citation of Giel Van Dooren and Soraya Zwahlen they should indicate in the text that it is a personal communication (i.e., not yet published).

**We thank the reviewers for this suggestion and for highlighting the editor as best suited to recommend a solution which we had followed by removing the comment about these data.**

S6 Fig caption does not list all the sequences for which alignments are shown, e.g., MPODD and SDH10. It also does not provide the names of the species included (shown as two-letter abbreviations in the figure). S7 Fig caption does not provide the name of the species designated as "Po" in one alignment.

**Thank you for noticing those errors which have been corrected now.**
